# Supplementary material for: Distinguishing Genetic Drift from Selection in Papillomavirus Evolution
Source: Viruses. 2023 Jul 26;15(8):1631. doi: 10.3390/v15081631 (PMC10458755; doi:10.3390/v15081631)
Supplement: Supplementary file 1 [file viruses-15-01631-s001.zip › S1. Regular expression patterns.pdf]

S1. Parameters recovered* with regular expression (RegEx) pattern-matching in unaligned genomes.**

Motif description	Coding strand	Non-coding Reverse Complement
CpG site	CG	CG
high affinity E2 site	ACC[GT]A\w{4}GGT	ACC\w{4}T[AC]GGT
non-canonical E2 site	ACAC\w{5}GGT	ACC\w{5}GTGT
APOBEC3 site	TC[AT]	[AT]GA
TLR9 stimulatory	[ACT][AG][AT]CGTT	AACG[AT][TC][TCG]
TLR9 suppressing	[AG]{2}CG[CT]{2}<br>(CC\w[GTA]{2}\w{2}GGG<br>[AT][GT]{2}[ACT]GGGG<br>TTAGGG	[GA]{2}CG[TC]{2}<br>CCC\w{2}[CTA]{2}\wGG<br>CCCC[AGT]CA{2}[AT]<br>CCCTAA
G quadruplexes	([G]{3,5}\w{1,12}){3}[G]{3,5}	([C]{3,5}\w{1,12}){3}[C]{3,5}
G duplexes	GGG\w{1,12}GGG	CCC\w{1,12}CCC

** relevant RegEx rules:

[GT] = a single nucleotide that is either G or T
\w = any single letter
{4} = 4 multiples of the immediately preceding portion of the expression
{3,7} = 3, 4, 5, 6 or 7 multiples of the immediately preceding portion of expression
Because two APOBEC3 sites can overlap (i.e., TCTCT,TCTCA) full discovery employed the iterative '?=TC[AT]' expression.

Python3 Code

```
import re
from Bio import SeqIO
from Bio.Seq import Seq

#obtain record from a GenBank formatted file
hpvfile=input("Genbank formatted file: ")
record = SeqIO.read(hpvfile, "genbank")

#obtain genome as string from record
genome=str(record.seq)

'''
using re.findall to find matching sites, the ?= finds overlapping motifs such that TCTCT is found to be 2 APOBEC3 sites not one
'''

#number of CpG sites both strands
numCpG=len(re.findall('(?=CG)', genome))
```

```
#number of APOBEC3 sites on the plus strand
numAPO3plus=len(re.findall('(?=TC[AT])',genome))

#number of APOBEC3 sites on the negative strand
numAPO3minus=len(re.findall('(?=[AT]GA)',genome))

#number of G quadruplexes both strands
numGquad= len(re.findall('(?=([gG]{3,5}\w{1,12}){3}[gG]{3,5} | ([cC]{3,5}\w{1,12}){3}[cC]{3,5})', genome))

#number of G duplexes both strands
numGdup= len(re.findall('(?=(GGG\w{1,12}GGG|CCC\w{1,12}CCC))', genome))

#number of high-affinity E2 binding sites both strands
numE2haff=len(re.findall('(?=ACC[GT]A\w{4}GGT|ACC\w{4}T[AC]GGT)', genome))

#number of non-canonical E2 binding sites both strands
numE2noncan= len(re.findall('(?=(ACAC\w{5}GGT)|(ACC\w{5}GTGT))',genome))

#number of TLR9 stimulating motifs both strands
numTLR9up= len(re.findall('(?= [ACT][AG][AT]CGTT|AACG[AT][TC][TCG])', genome))

#number of TLR9 suppressing motifs both strands
numTLR9down=
len(re.findall('(?=[AG]{2}CG[CT]{2}|[GA]{2}CG[TC]{2}|(CC\w[GTA]{2}\w{2}GGG|CCC\w{2}[CTA]{2}\wGG|[AT][GT]{2}[ACT]G
GGG|CCCC[AGT]CA{2}[AT]|TTAGGG|CCCTAA)', genome))

'''
 additional parameters that were not found with regular expression pattern matching included base composition,
palindromes, inverted repeats, large duplicated regions in forward and reverse orientation).
'''

Acont=genome.count('A')/len(genome)
Ccont=genome.count('C')/len(genome)
Gcont=genome.count('G')/len(genome)
Tcont=genome.count('T')/len(genome)
GCcont=Gcont+Ccont

#find inverted repeats min size 5 each side, max separation 45, not palindromes
numinverts=0
for x in range (0,len(genome)-10):
    aseq=Seq(genome[x:x+5])
    restofgenome=genome[x+5:]
    rcseq=aseq.reverse_complement()
    thisrptdist= restofgenome.find(str(rcseq))
    if thisrptdist<50 and thisrptdist>10:
        numinverts+=1

#find perfect palindromes min size 8
numpalind=0
for x in range (0,len(genome)-8):
    aseq=Seq(genome[x:x+8])
    rcseq=aseq.reverse_complement()
    if aseq==rcseq:
      numpalind+=1

'''
find nonoverlapping matching sequences forward and revcomp at least 14 nt long and 50 nt apart
'''
numrpts=0
numrcrpts=0
for x in range (0,len(genome)-78):
    aseq=Seq(genome[x:x+14])
    smallgenome=genome[x+64:]
```

```
numrpts+=len(re.findall(str(aseq),smallgenome))
aseq=aseq.reverse_complement()
numrcrpts+=len(re.findall(str(aseq),smallgenome))
```
