# Supplementary material for: Distinguishing Genetic Drift from Selection in Papillomavirus Evolution
Source: Viruses. 2023 Jul 26;15(8):1631. doi: 10.3390/v15081631 (PMC10458755; doi:10.3390/v15081631)
Supplement: Supplementary file 1 [file viruses-15-01631-s001.zip › S10. Correlated Phylogenetic Independent Contrasts .pdf]

**S10. Correlated Phylogenetic Independent Contrasts of DNA Sequence Motifs*.**

| | R | Q |
|---|---|---|
| ***Alphapapillomavirus*** | | |
| CpG | | |
| G duplexes | 0.700 | <0.001 |
| Inverted repeats | -0.343 | 0.004 |
| Inverted repeats + palindromes | -0.378 | 0.001 |
| TLR9 | 0.396 | 0.001 |
| **Low Risk 1** | | |
| CpG | | |
| G duplexes | 0.750 | 0.007 |
| **Low Risk 2** | | |
| CpG | | |
| G duplexes | 0.600 | 0.004 |
| APOBEC3 on + strand | 0.546 | 0.008 |
| % APOBEC3 on – strand | -0.636 | 0.001 |
| TLR9 | | |
| APOBEC3 | 0.643 | 0.003 |
| APOBEC3 on + strand | 0.688 | 0.001 |
| APOBEC3 on + strand | | |
| APOBEC3 on – strand | 0.696 | <0.001 |
| Inverted repeats + palindromes | | |
| APOBEC3 on – strand | -0.534 | 0.023 |
| **High Risk Human** | | |
| CpG | | |
| APOBEC3 | -0.644 | 0.001 |
| ***Alphapapillomavirus 5 + Alphapapillomavirus 6*** | | |
| G quadruplexes | | |
| E2 sites | 0.521 | 0.006 |
| APOBEC3 on + strand | 0.658 | <0.001 |
| ***Alphapapillomavirus 7*** | | |
| APOBEC3 | | |
| CpG | -0.593 | <0.001 |
| TLR9 | 0.560 | 0.001 |
| Non-canonical E2 sites | - 0.583 | <0.001 |
| G duplexes | -0.703 | <0.001 |
| ***Alphapapillomavirus 9*** | | |
| TLR9 | | |
| E2 sites | 0.540 | 0.001 |
| APOBEC3 | -0.489 | 0.003 |
| Inverted repeats | 0.428 | 0.003 |
| G quadruplexes | -0.465 | 0.003 |

*excluding autocorrelated pairs (e.g., G duplexes/G quadruplexes).
