# Supplementary material for: Distinguishing Genetic Drift from Selection in Papillomavirus Evolution
Source: Viruses. 2023 Jul 26;15(8):1631. doi: 10.3390/v15081631 (PMC10458755; doi:10.3390/v15081631)

S3. Significant changes in selection strength found with RELAX
K=selection strength, LR=likelihood ratio, https://www.datamonkey.org/relax/

LR2: selection <u>relaxation</u> was significant (K = 0.63, LR = 50.72, p = 0.000) as compared to *Alphapapillomavirus*

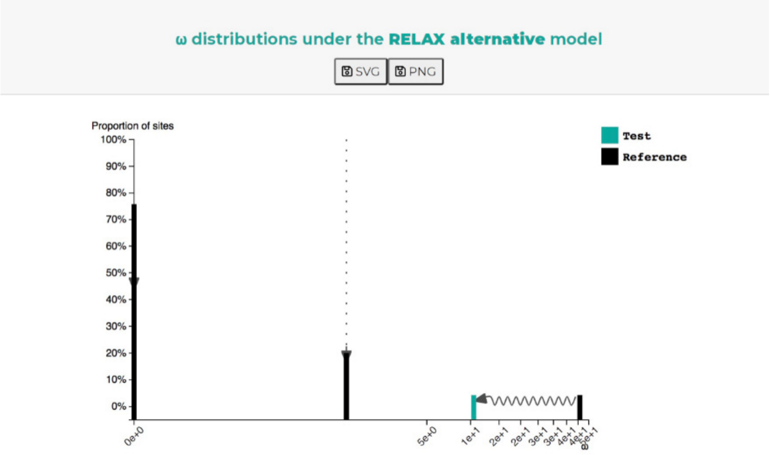

Alpha12: selection <u>intensification</u> was significant (K = 1.21, LR = 40.39, p = 0.000) as compared to *Alphapapillomavirus*

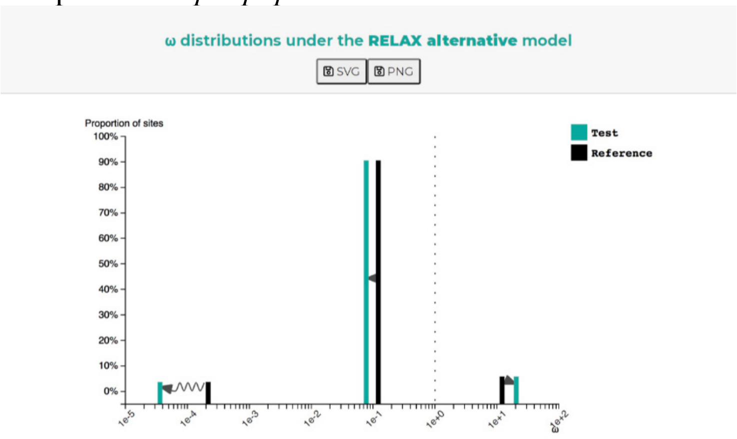

Alpha12: selection <u>intensification</u> was significant (K = 1.42, LR = 100.12, p = 0.000) as compared to other mucosal clade close relatives (HR+LR1)

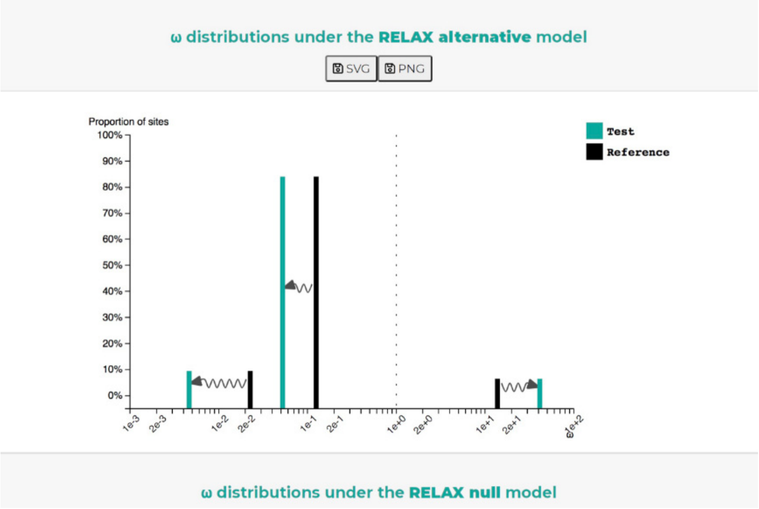

Supplement: Supplementary file 1 [file viruses-15-01631-s001.zip › S3. Selection strength RELAX.pdf]
