# Supplementary material for: Distinguishing Genetic Drift from Selection in Papillomavirus Evolution
Source: Viruses. 2023 Jul 26;15(8):1631. doi: 10.3390/v15081631 (PMC10458755; doi:10.3390/v15081631)
Supplement: Supplementary file 1 [file viruses-15-01631-s001.zip › S9. HPVAlpha9 nt changes.pdf]

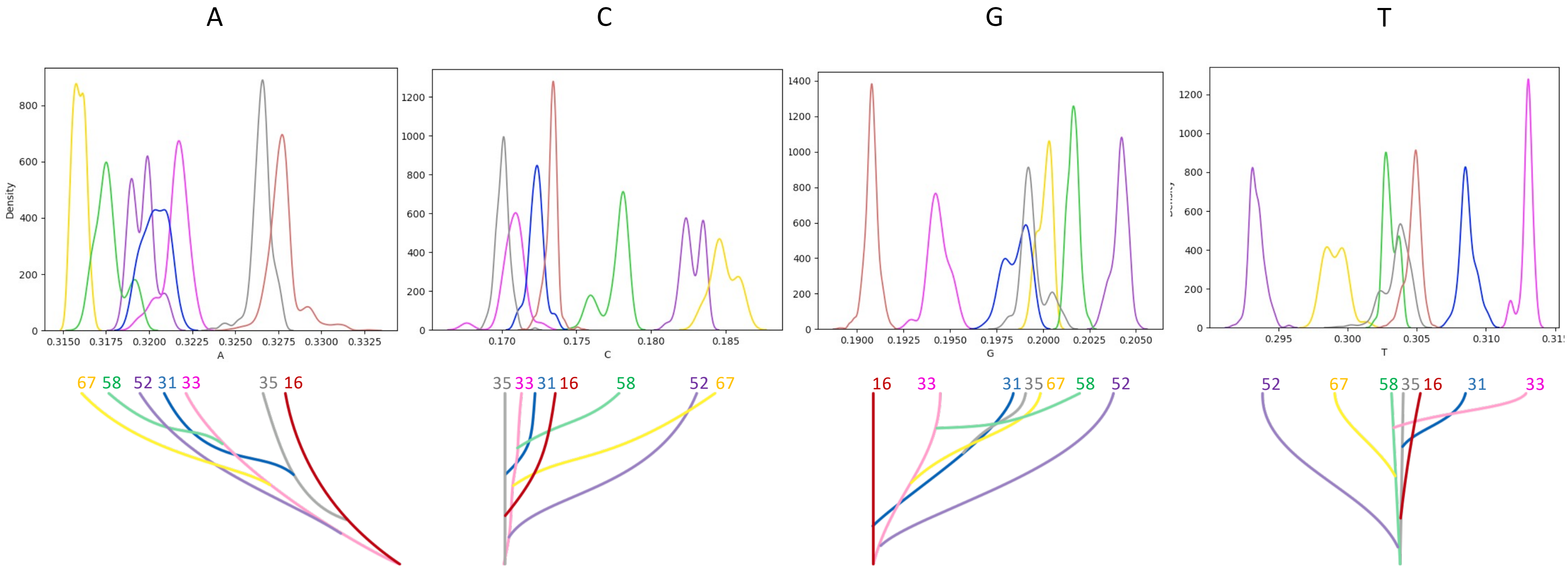

Base compositions from 1,278 complete *Alphapapillomavirus 9* genomes standardized to equal representation by HPV type superimposed on phylogenetic trajectories from the directional-drift (nhPhyML) estimate of the ancestral composition for the species.

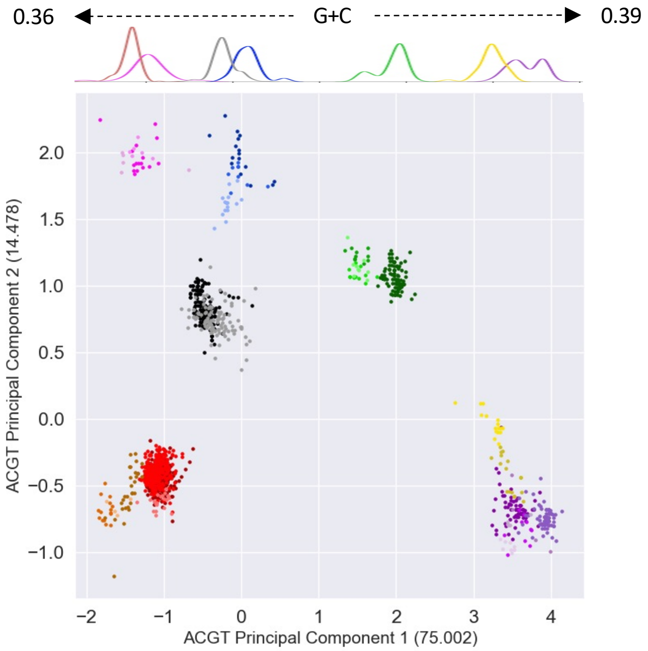

G+C content superimposed on the first principal component of base compositions (see Figure 5) from 1,278 complete *Alphapapillomavirus 9* genomes.

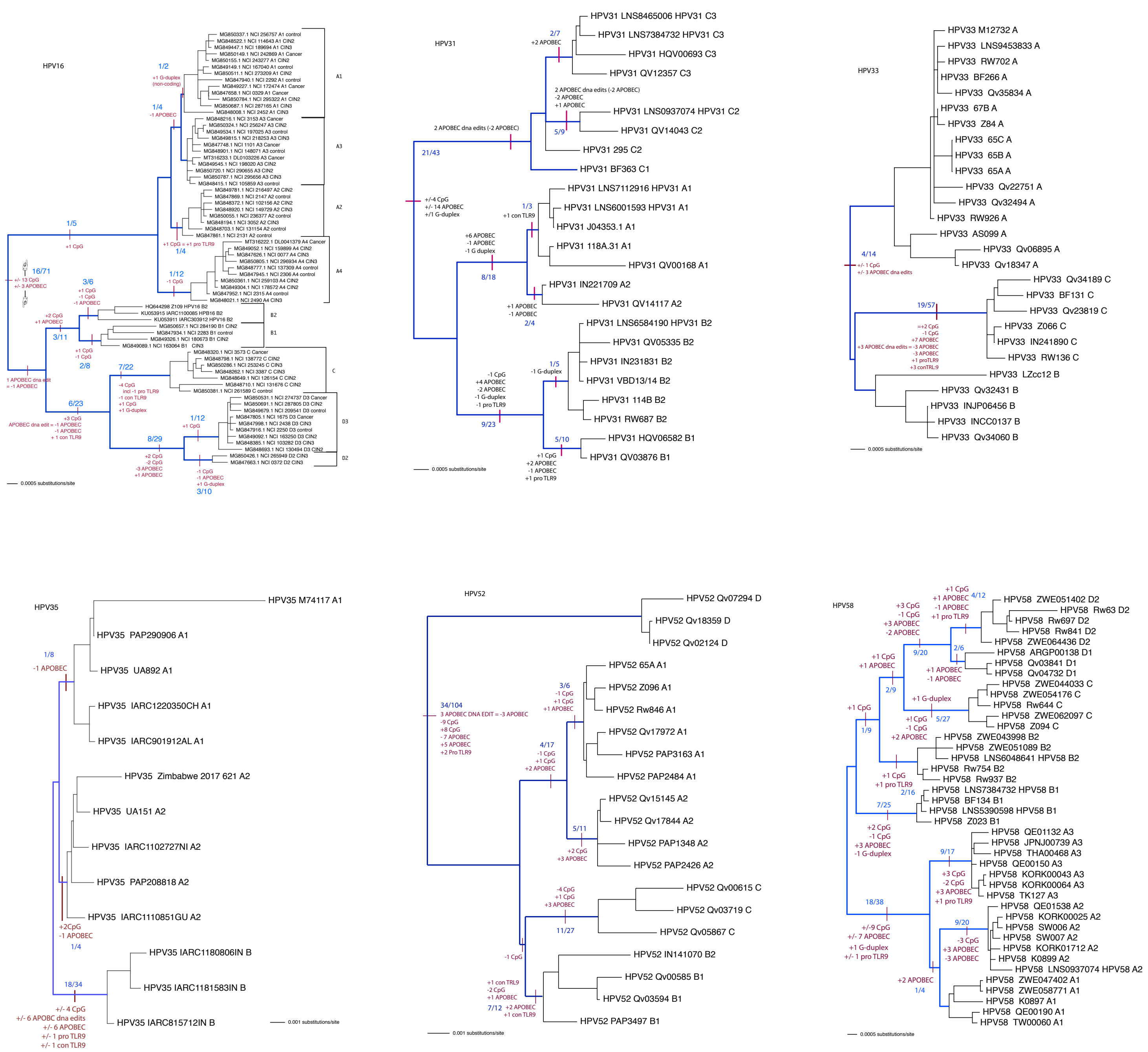

Maximum Likelihood site-by-site direct examination of ancestral nucleotide site transformations from suites of aligned complete genomes across lineages and variants within alpha9 types. Number of nucleotide transformations corresponding to gains and losses of CpG and APOBEC3 sites (in red) as well as the ratio of their sum to the total of all nucleotide transformations (in blue) are denoted on each branch subtending each distinct subtype lineage.
