# Supplementary figures and images for: Distinguishing Genetic Drift from Selection in Papillomavirus Evolution
Source: Viruses. 2023 Jul 26;15(8):1631. doi: 10.3390/v15081631 (PMC10458755; doi:10.3390/v15081631)

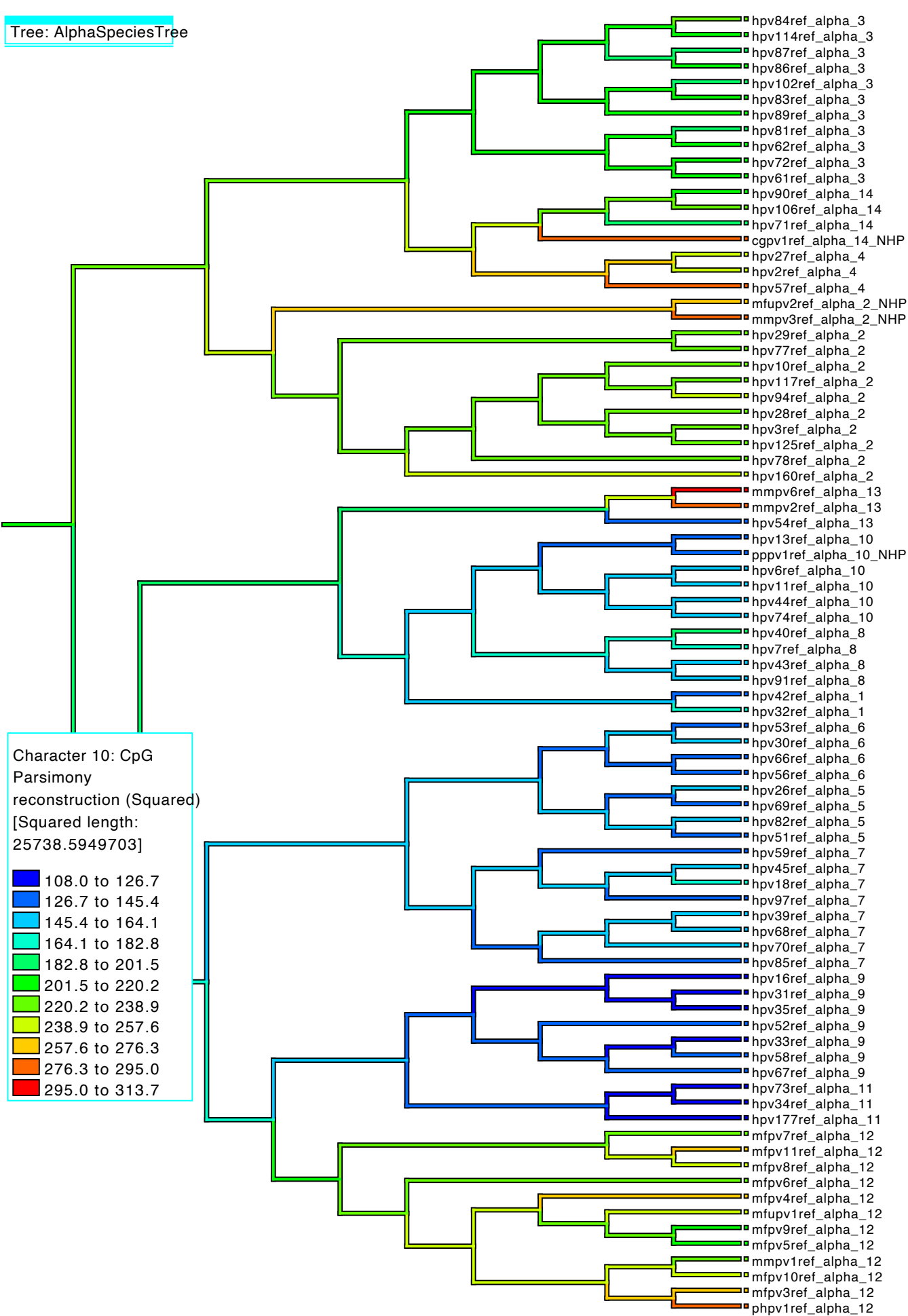

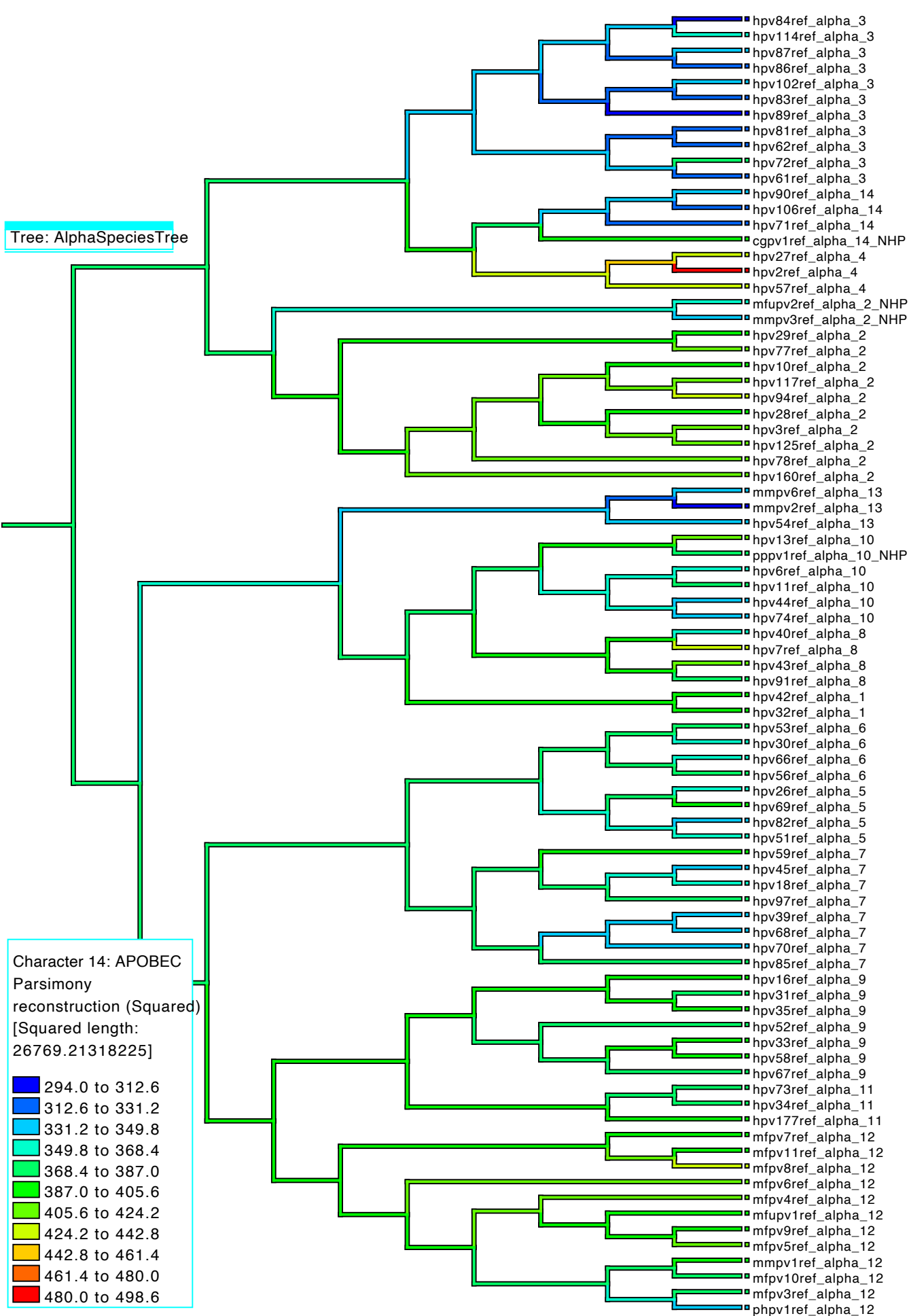

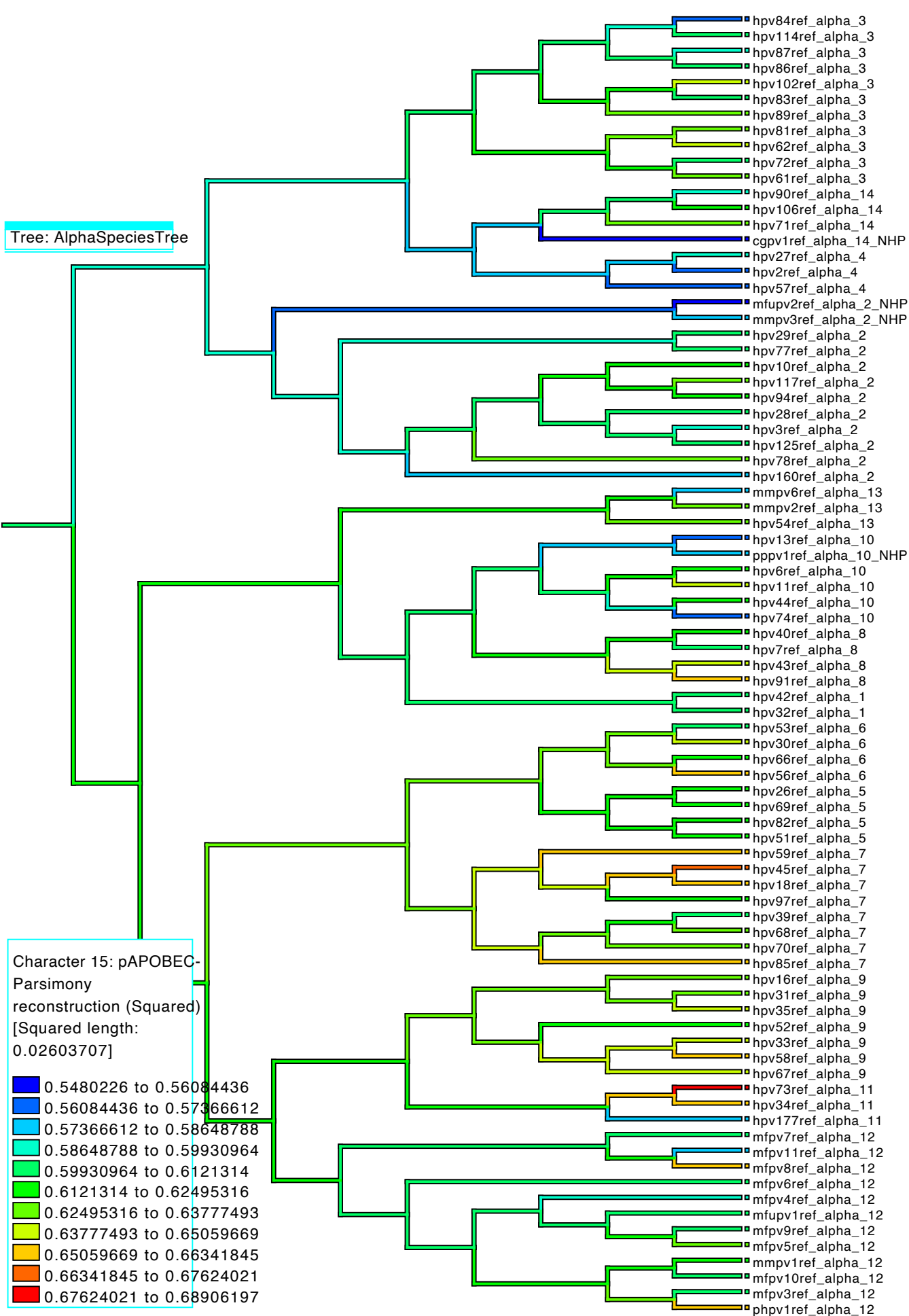

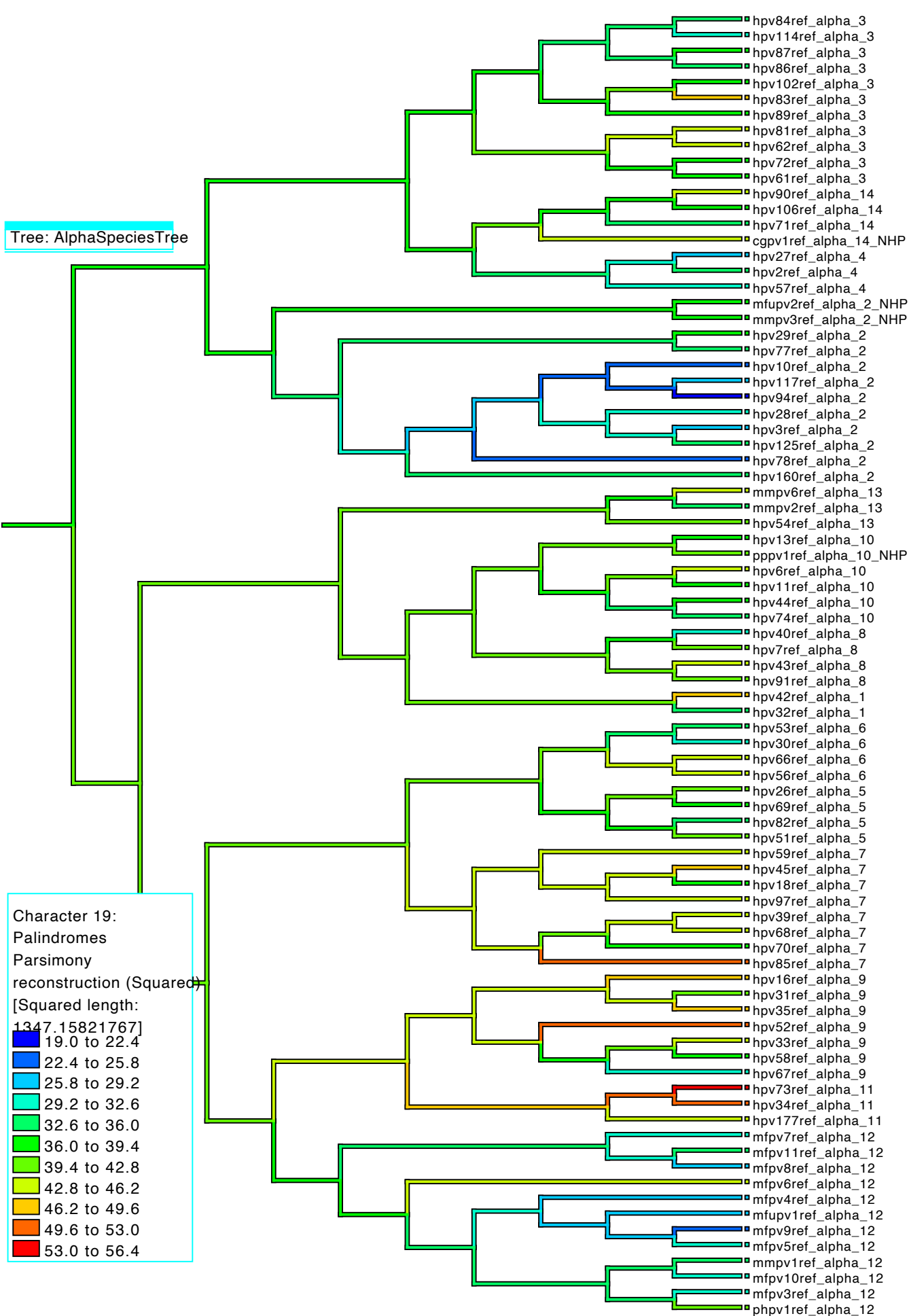

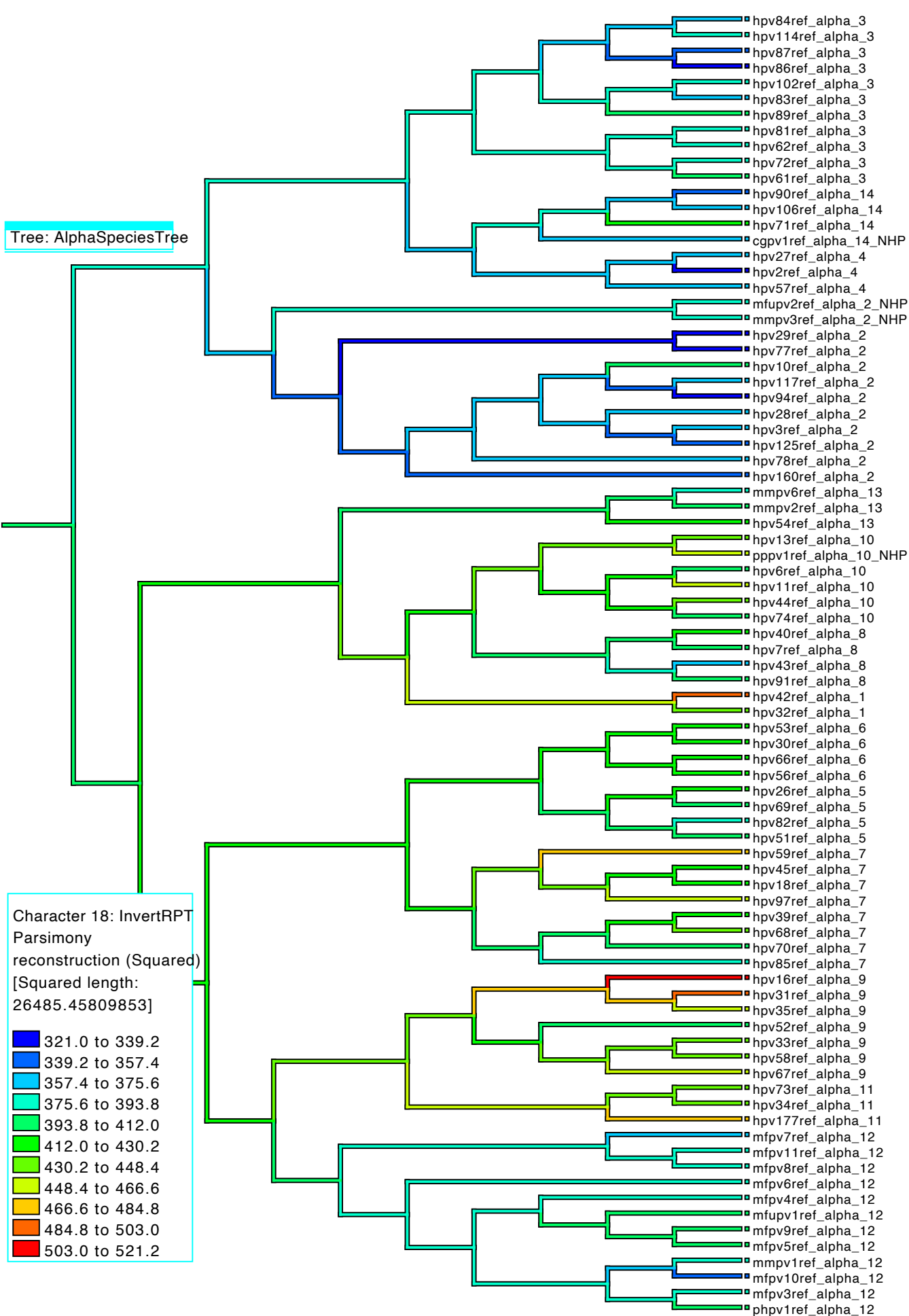

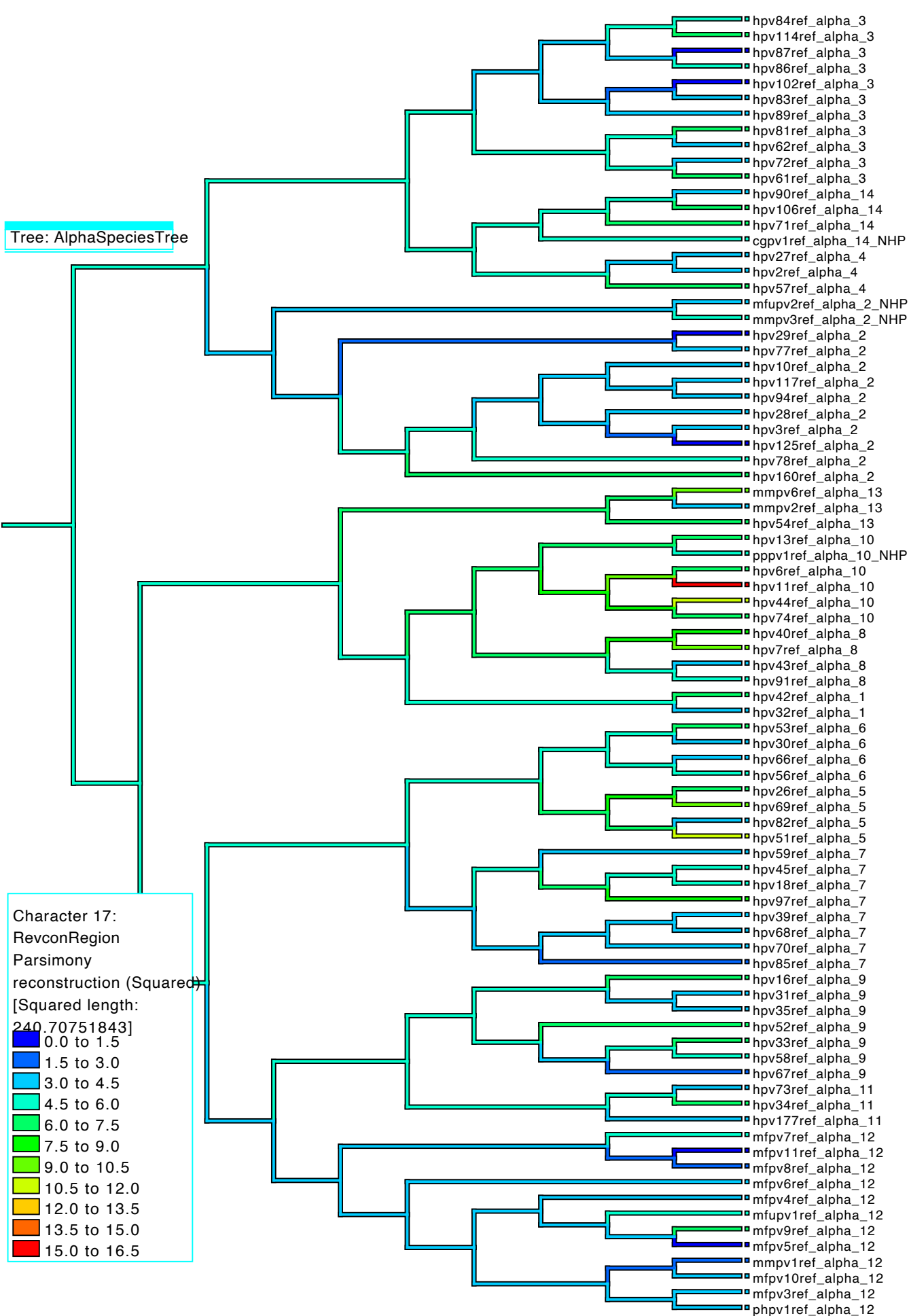

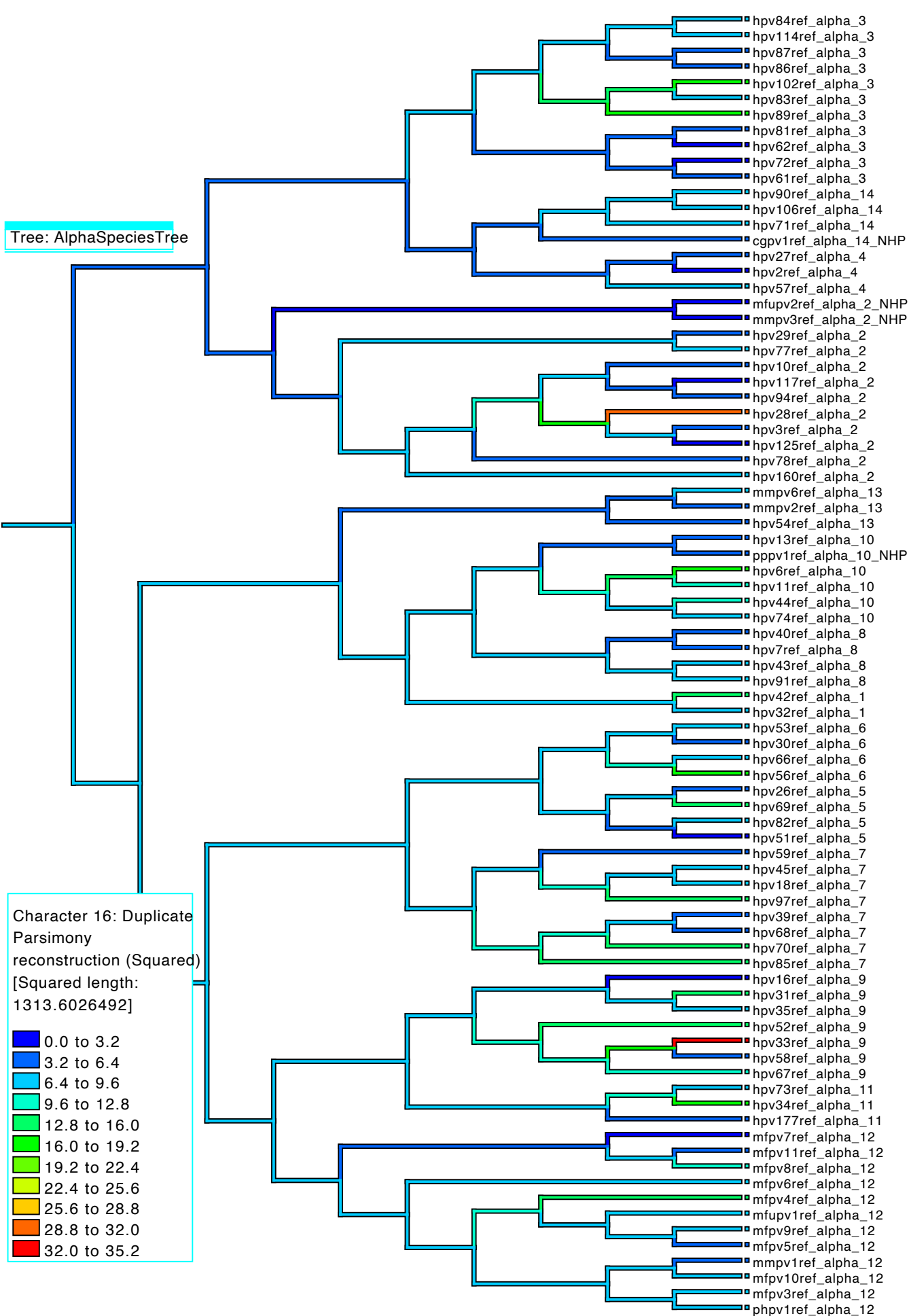

Character 13: TLR9cg  
Parsimony  
reconstruction (Squared)  
[Squared length:  
264.9041102]

|              |
|--------------|
| 0.0 to 1.5   |
| 1.5 to 3.0   |
| 3.0 to 4.5   |
| 4.5 to 6.0   |
| 6.0 to 7.5   |
| 7.5 to 9.0   |
| 9.0 to 10.5  |
| 10.5 to 12.0 |
| 12.0 to 13.5 |
| 13.5 to 15.0 |
| 15.0 to 16.5 |

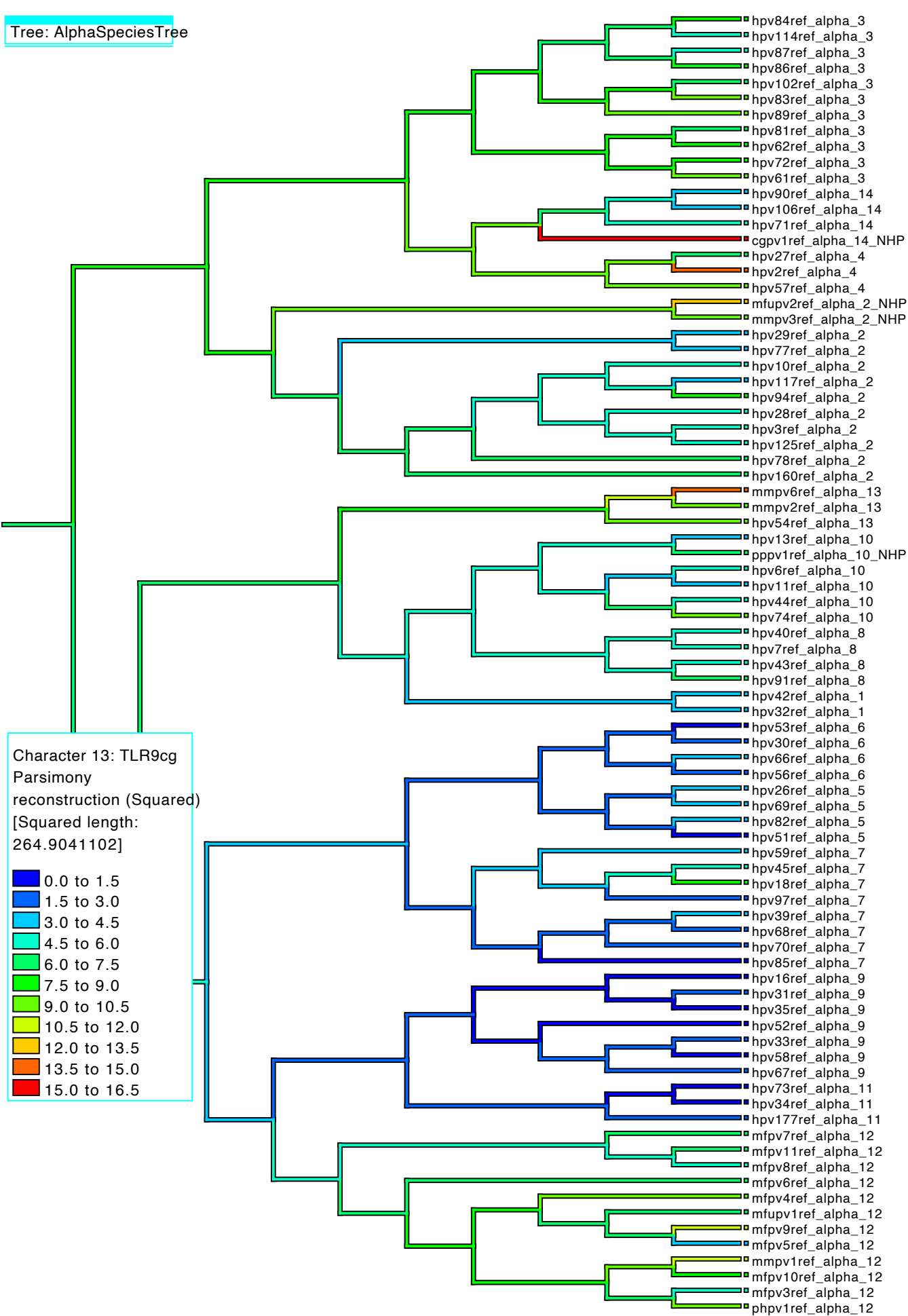

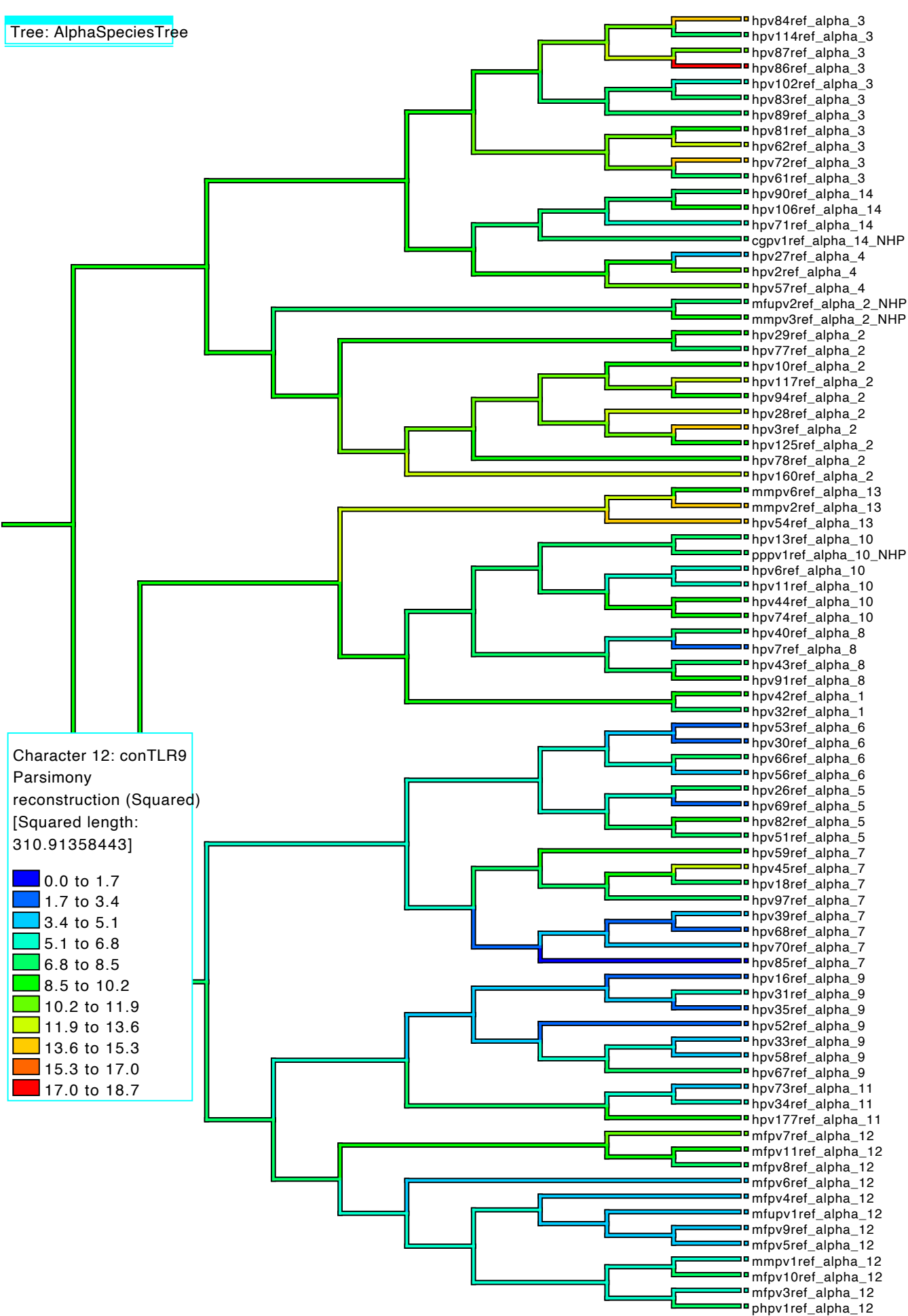

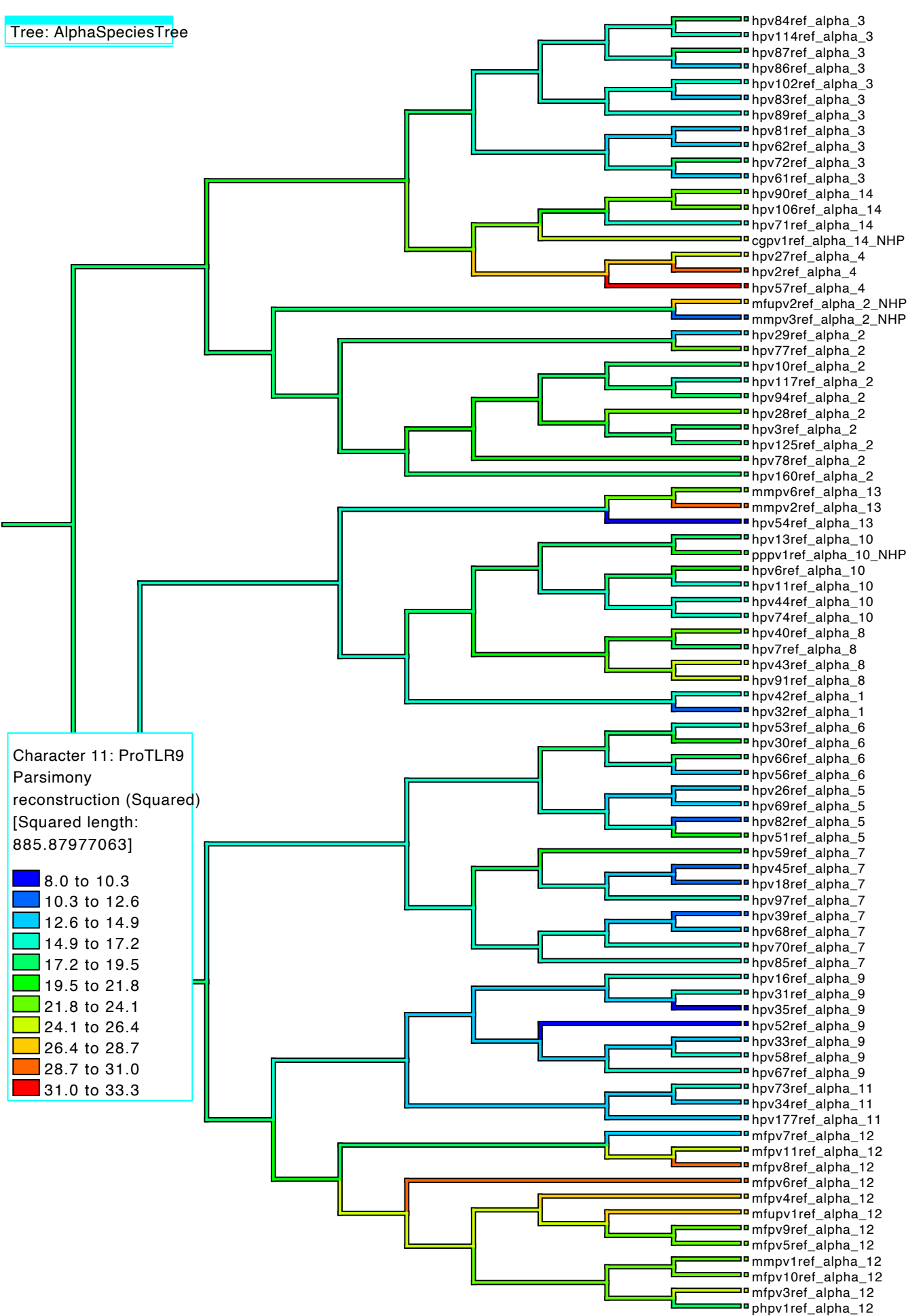

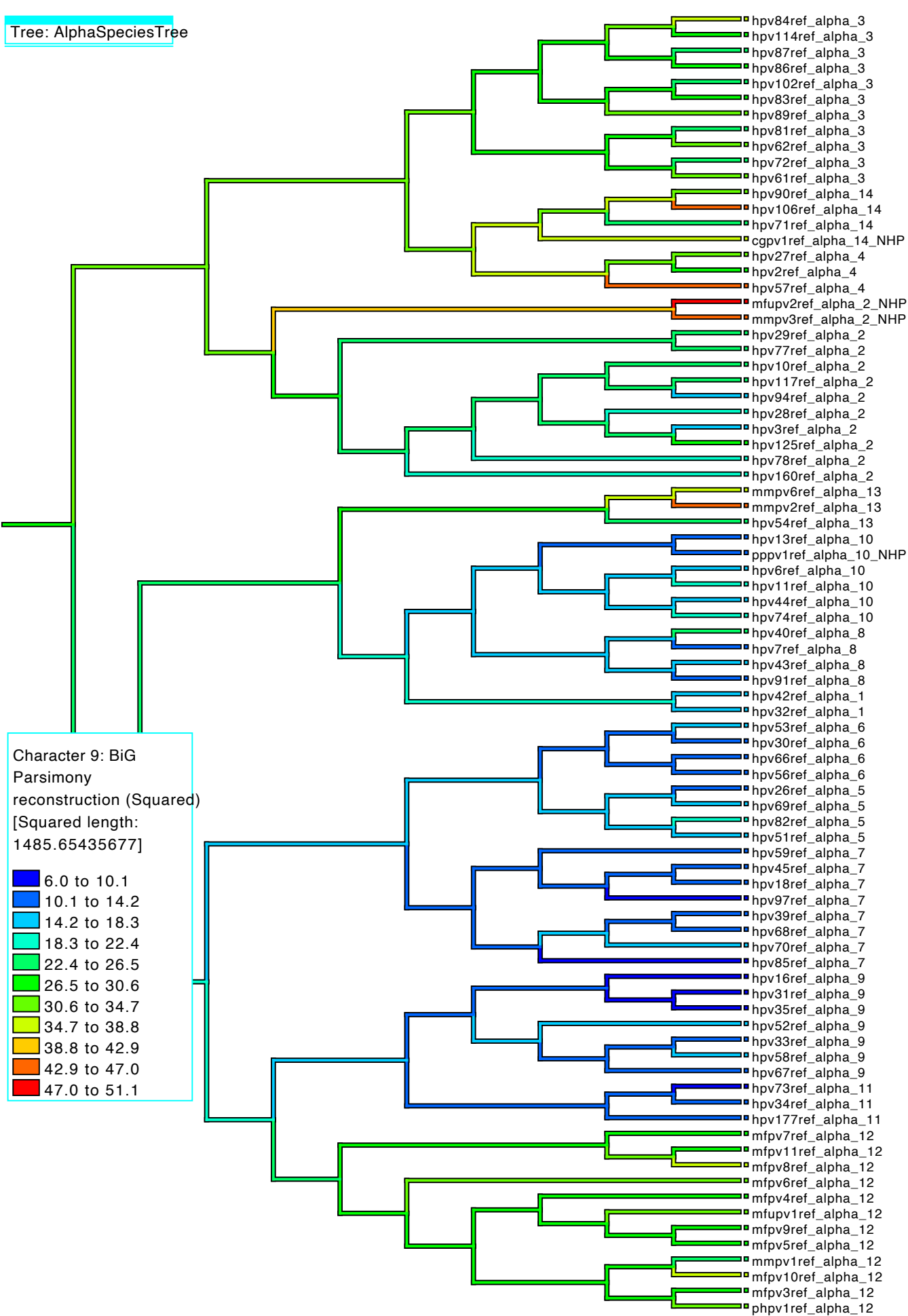

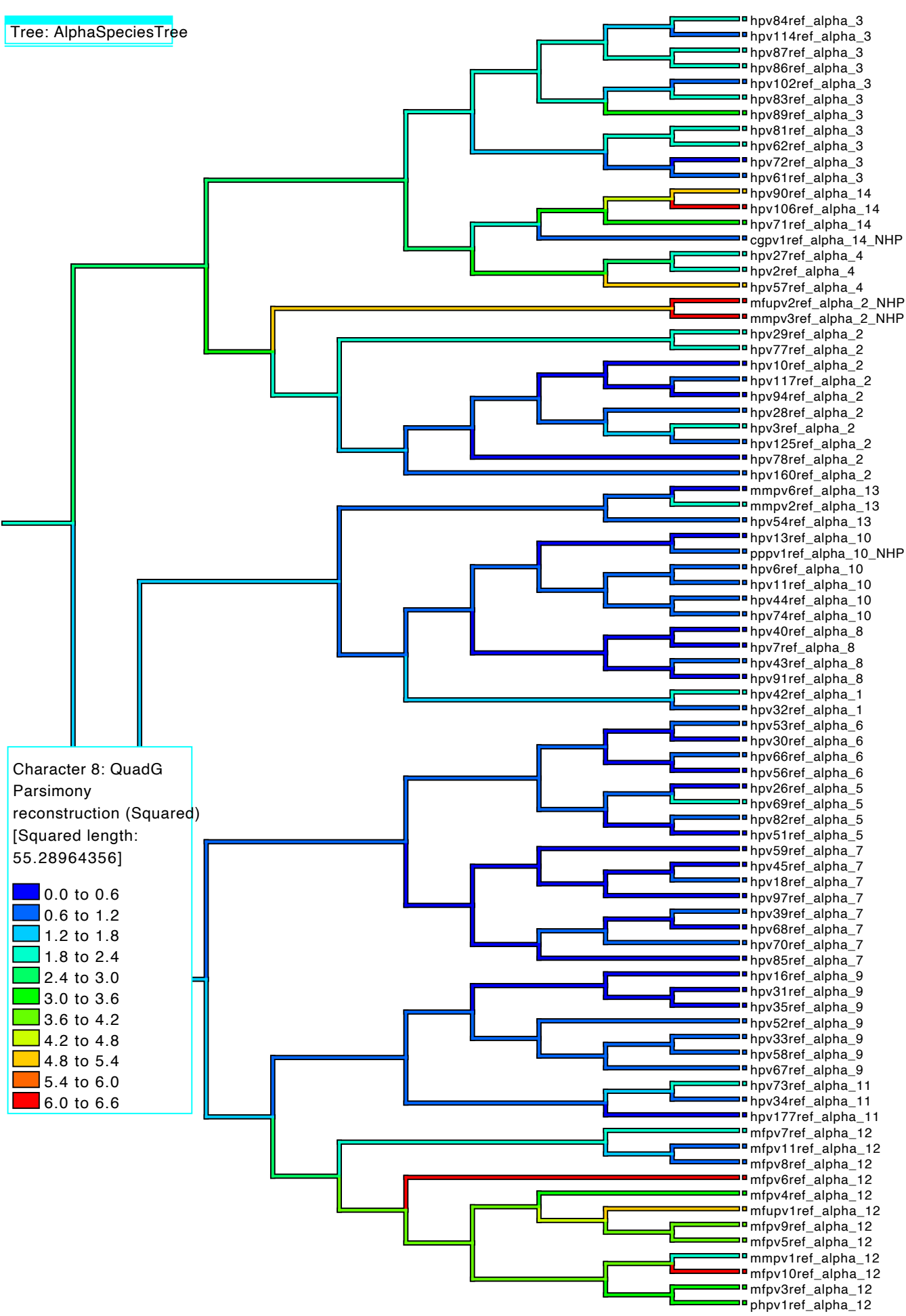

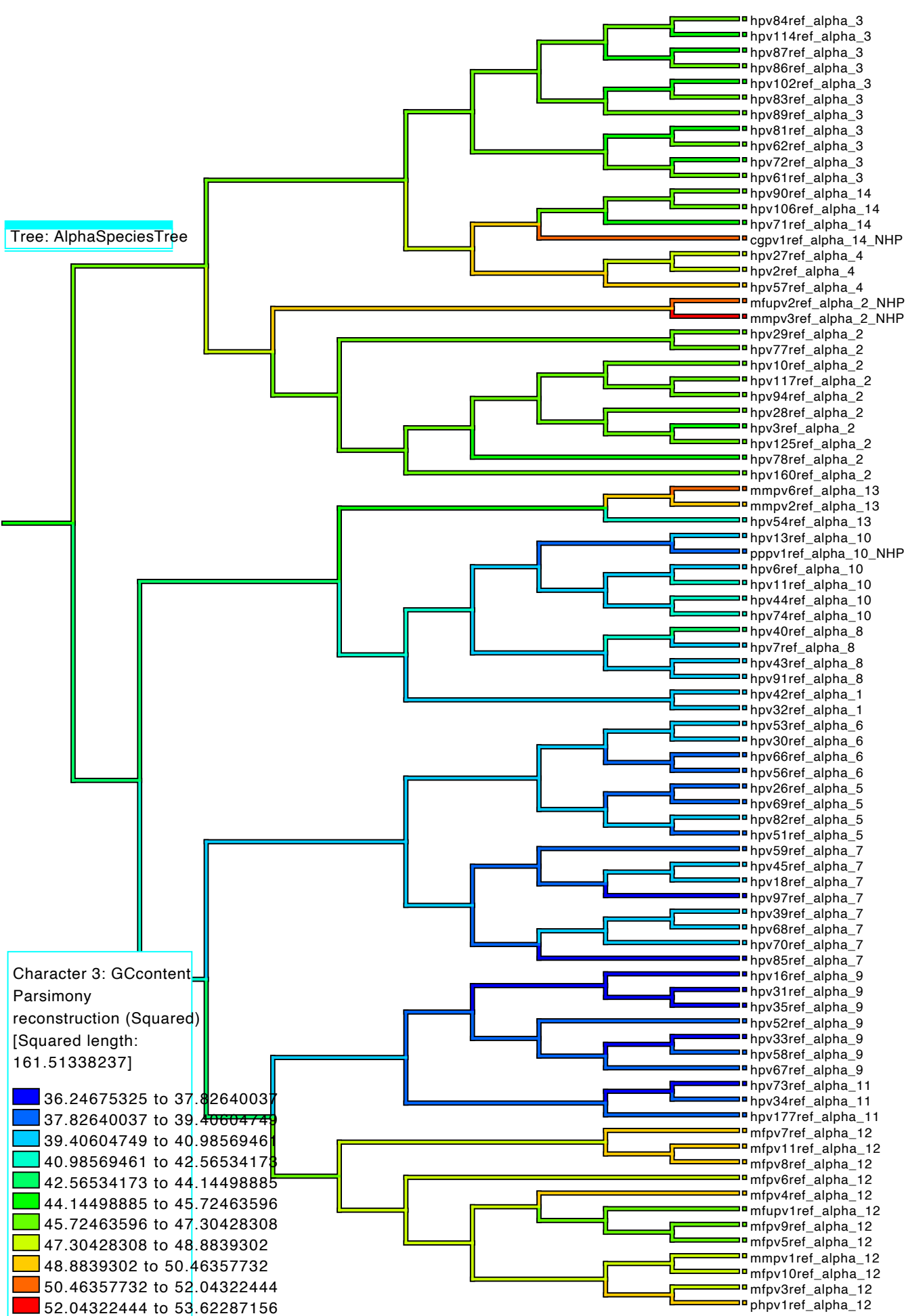

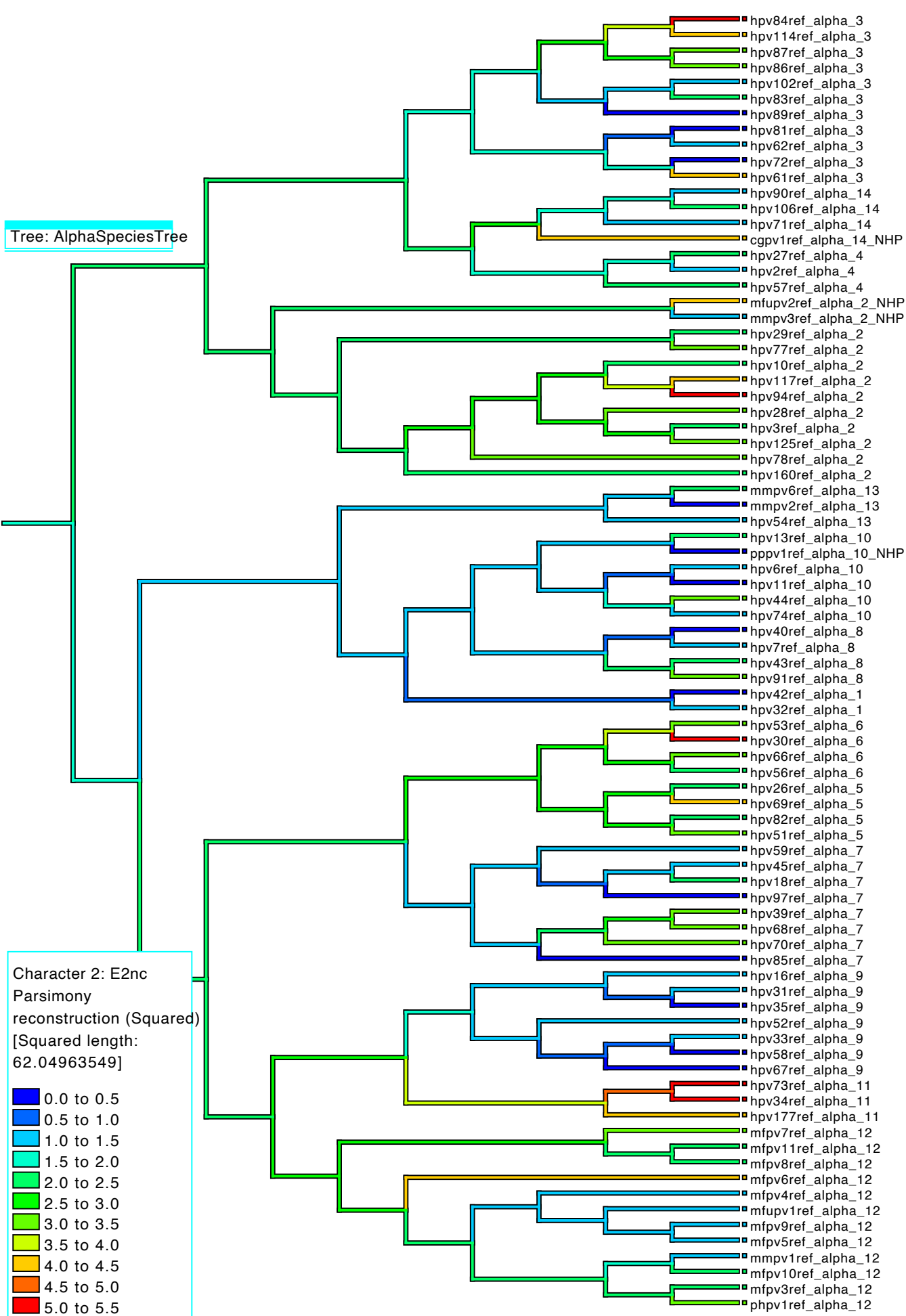

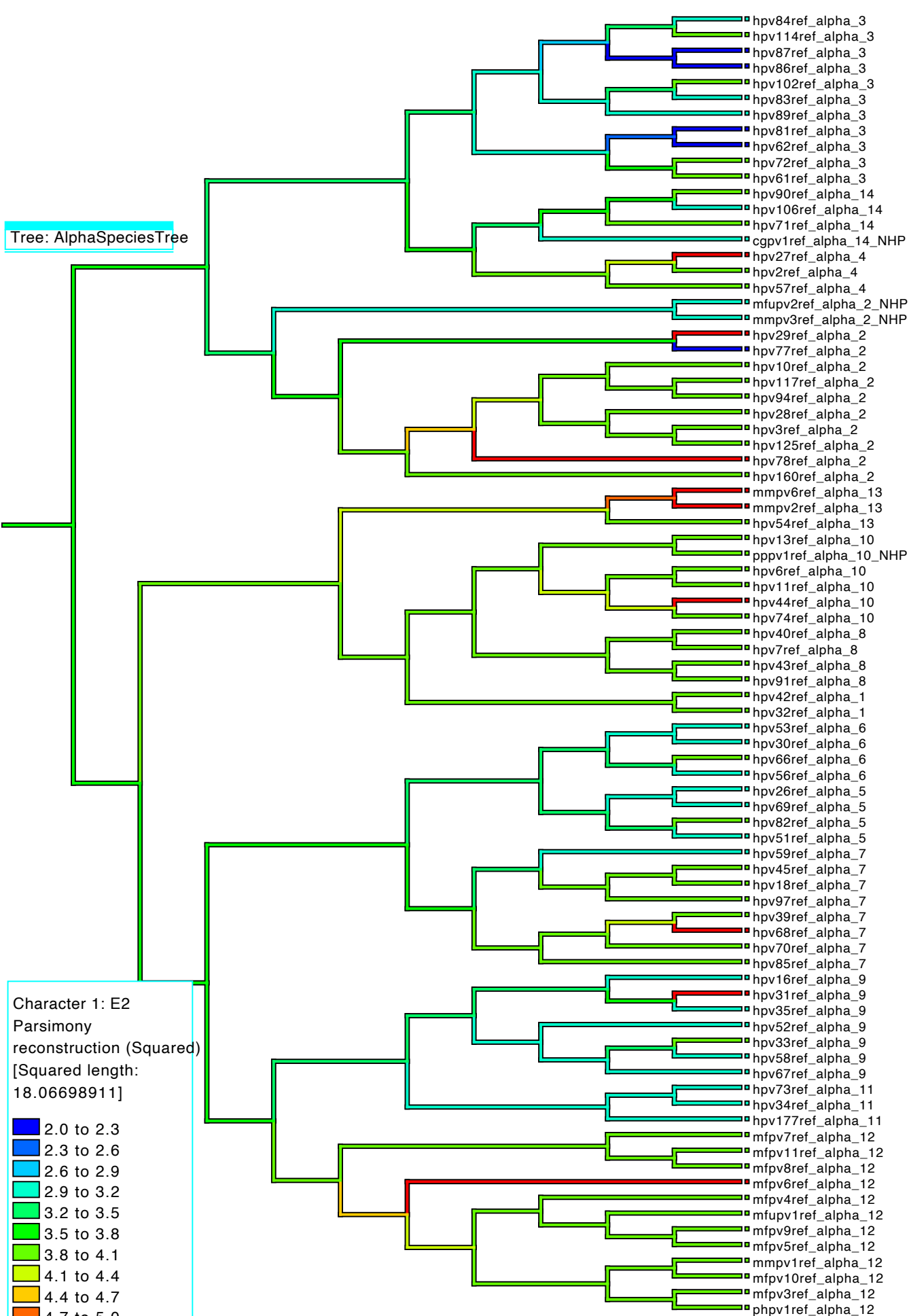

Supplement: Supplementary file 1 [file viruses-15-01631-s001.zip › S4. HPV Reference Types Ancestral Reconstructions.pdf]

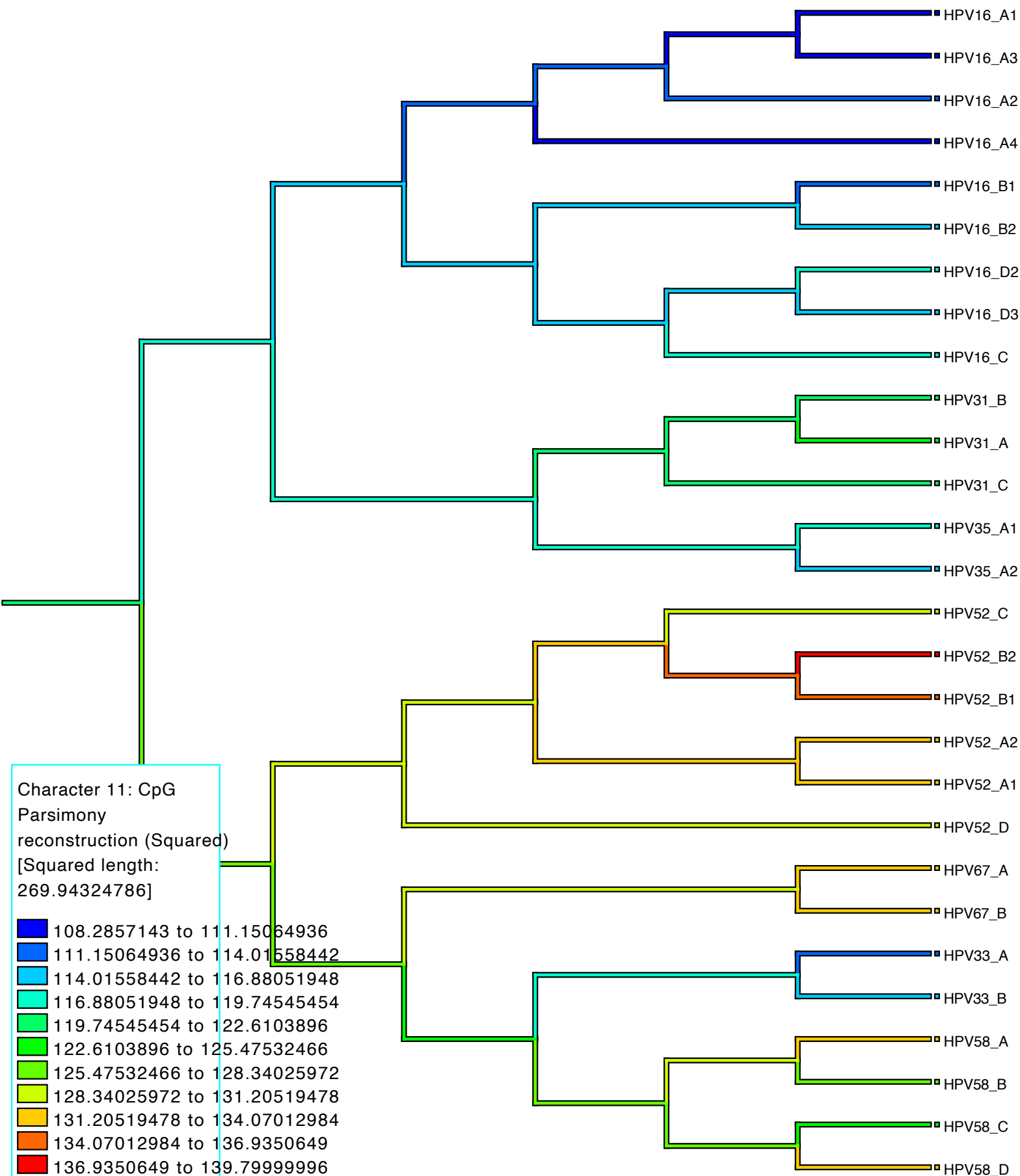

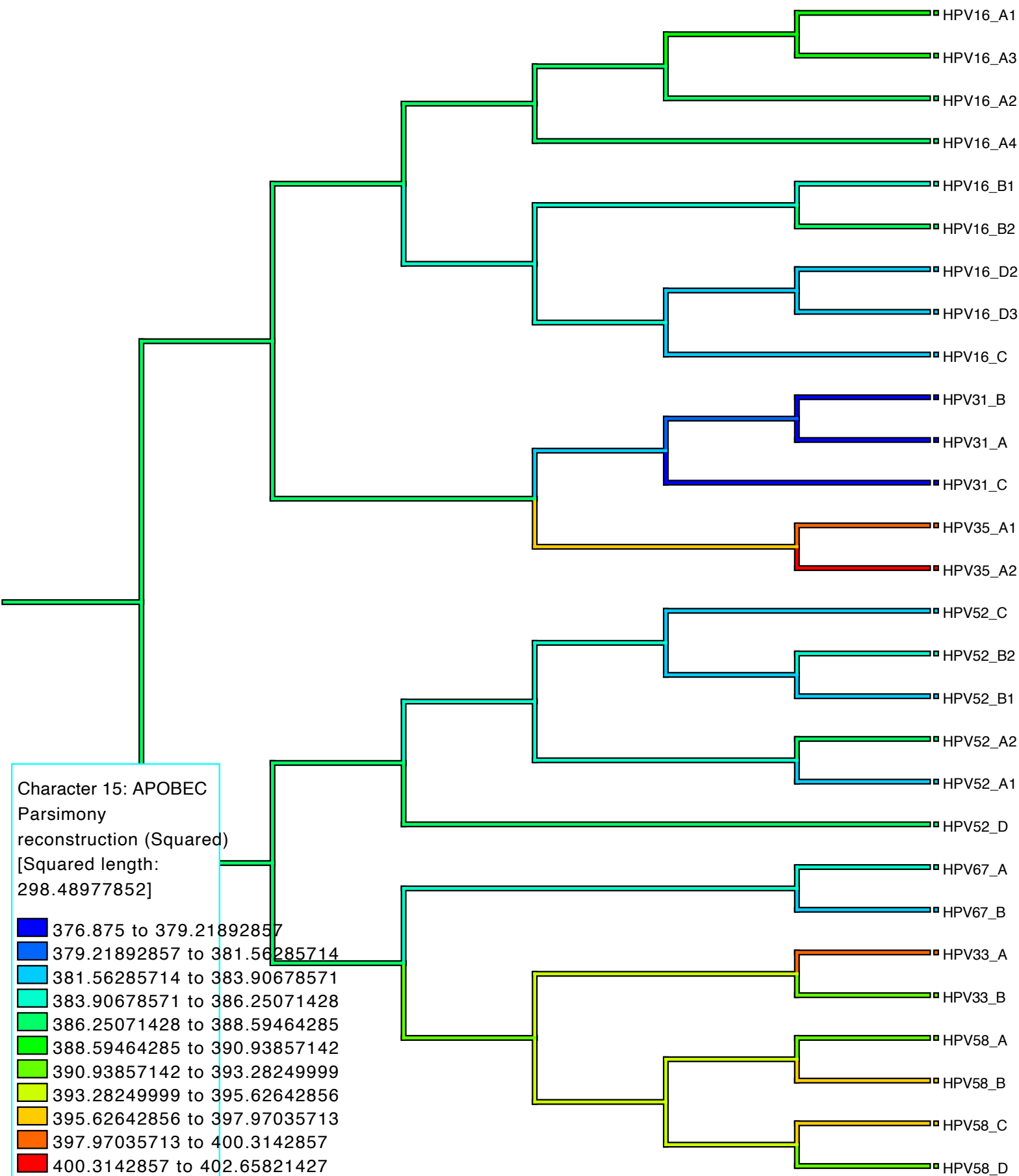

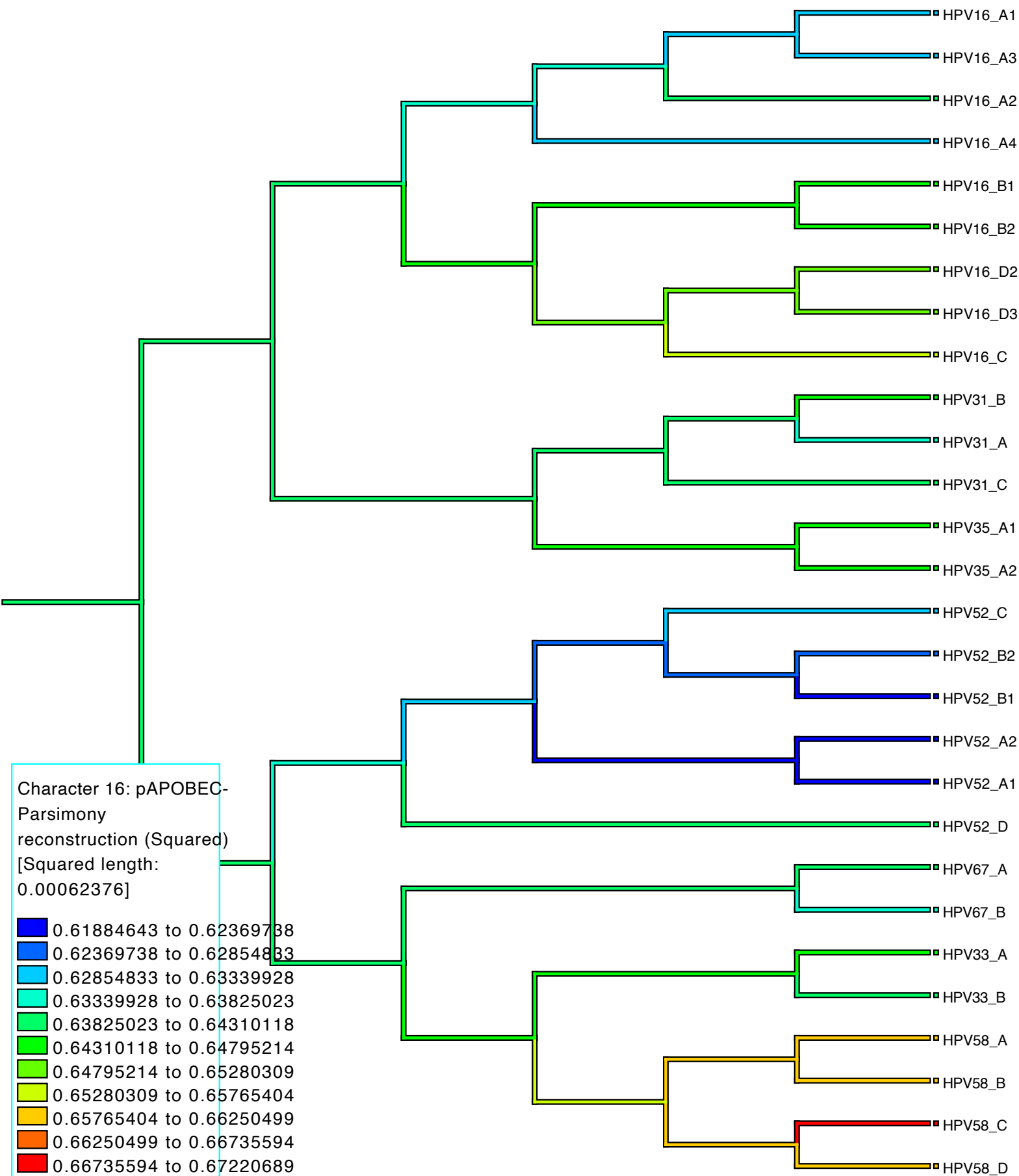

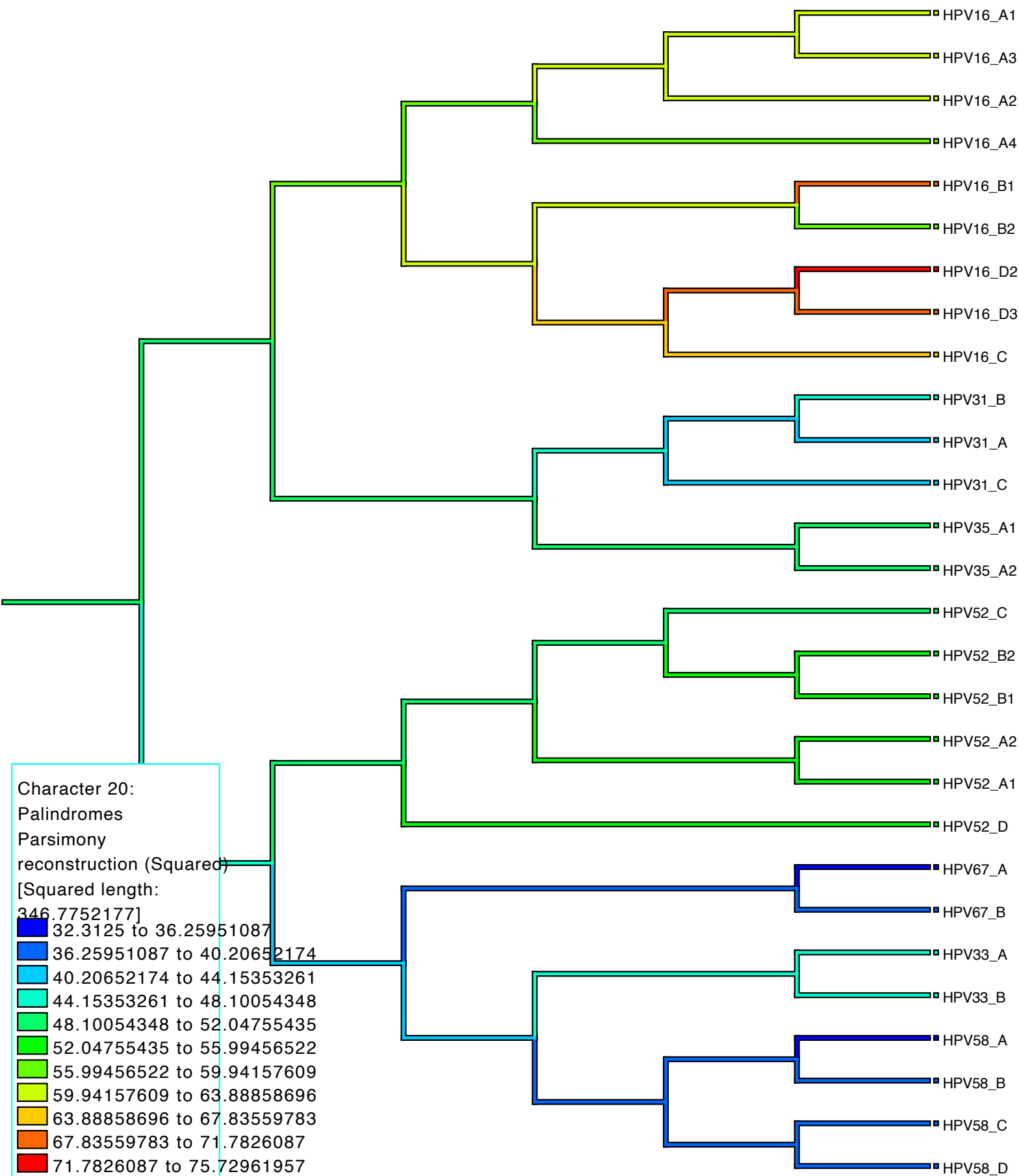

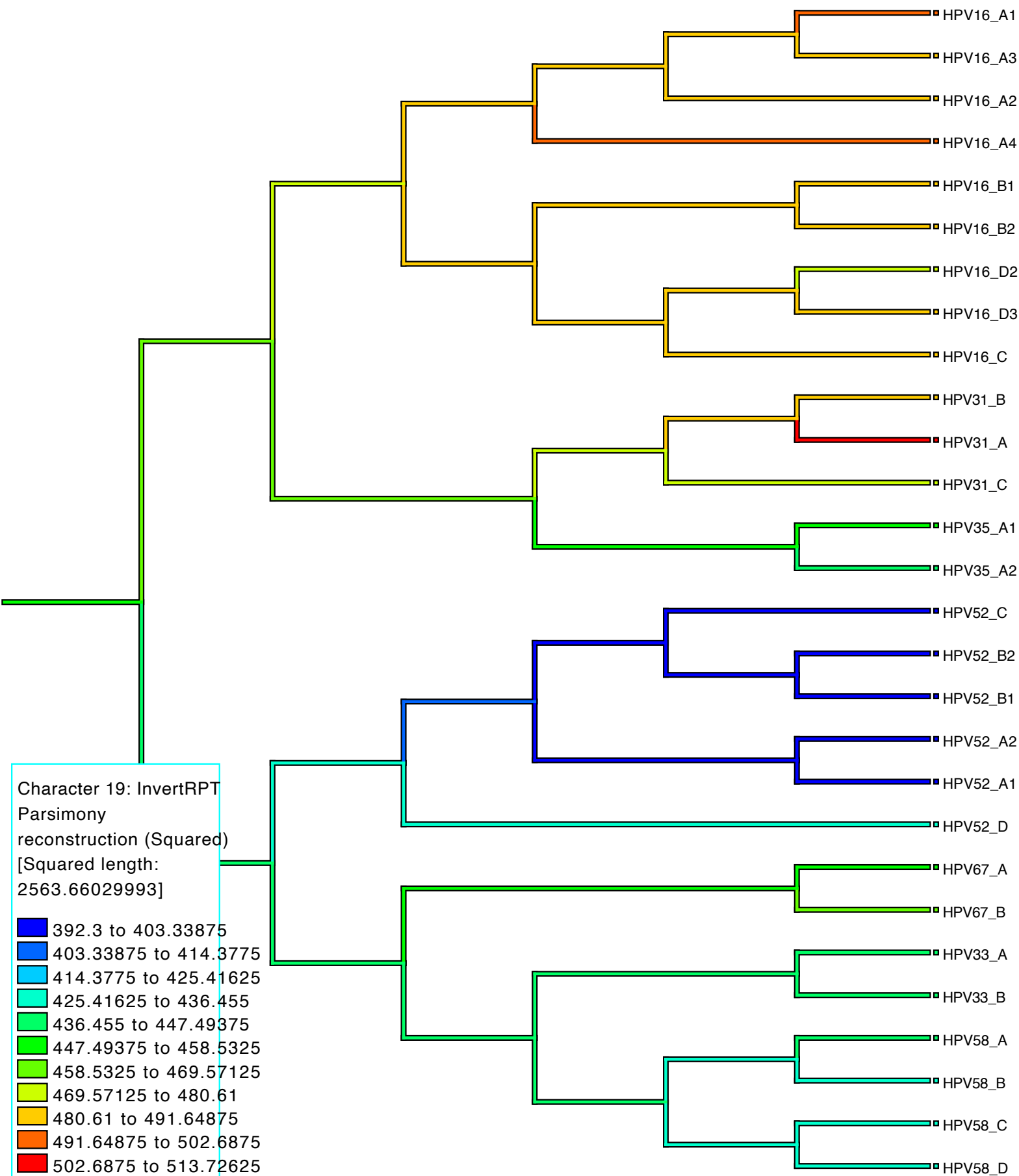

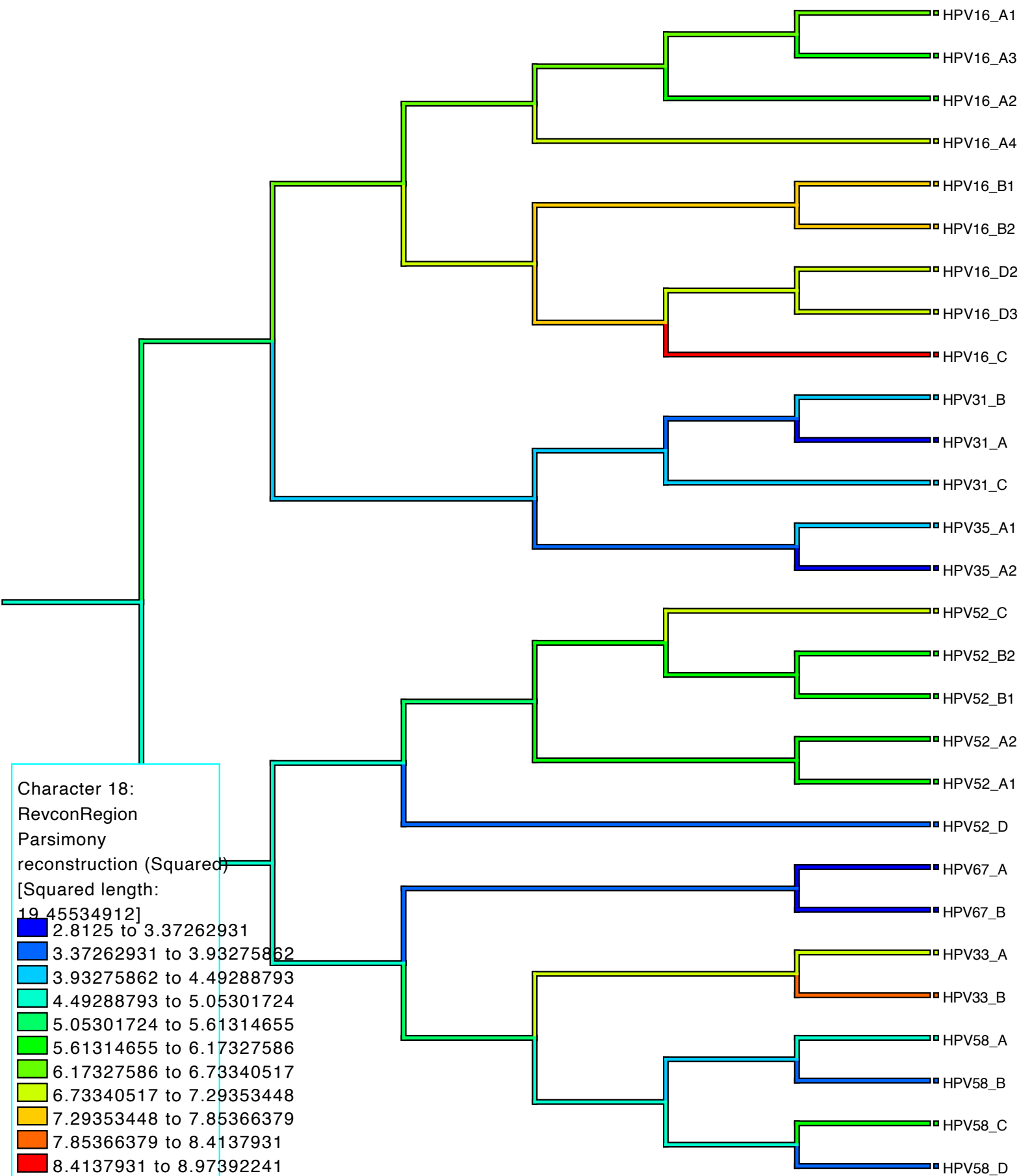

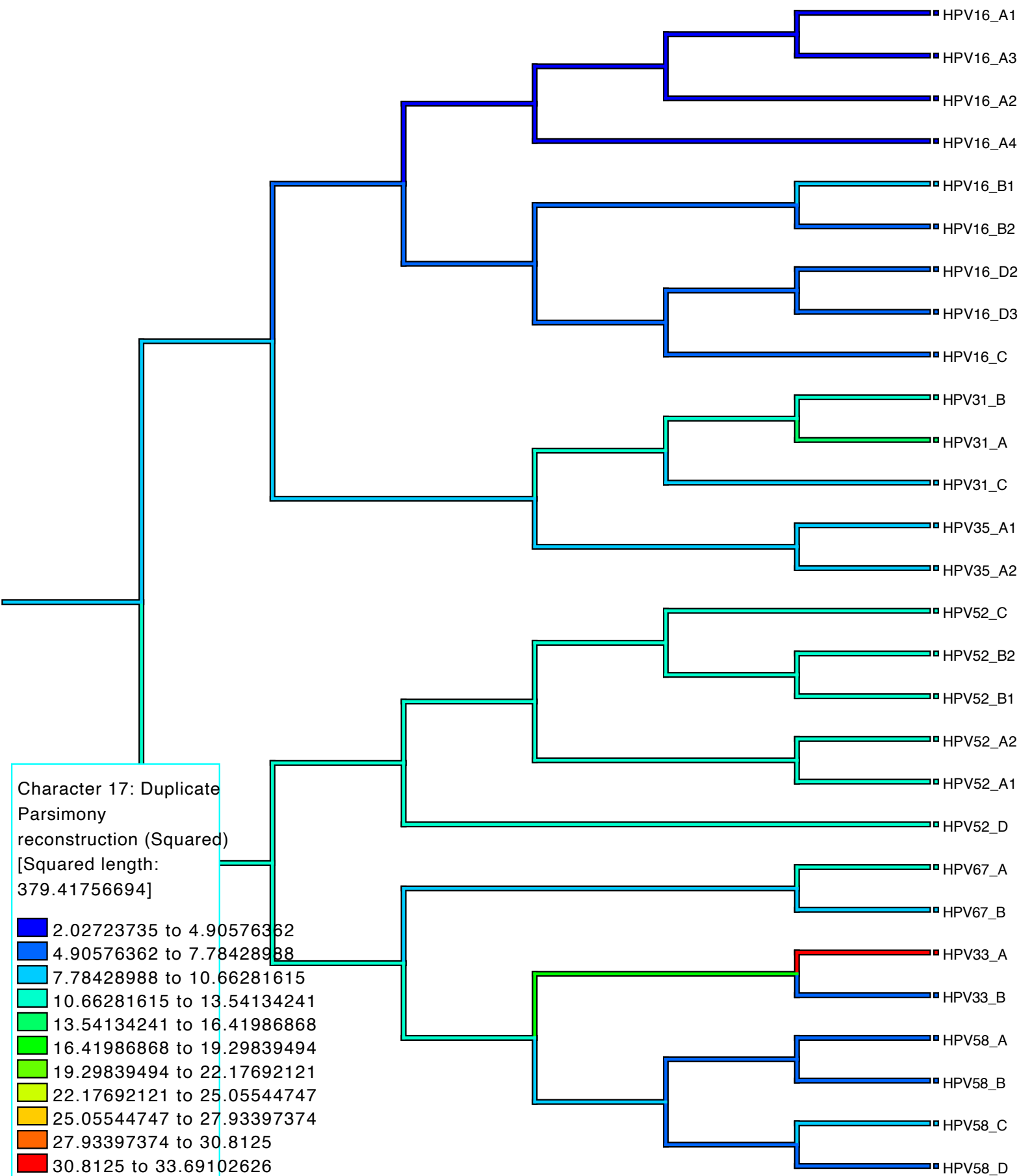

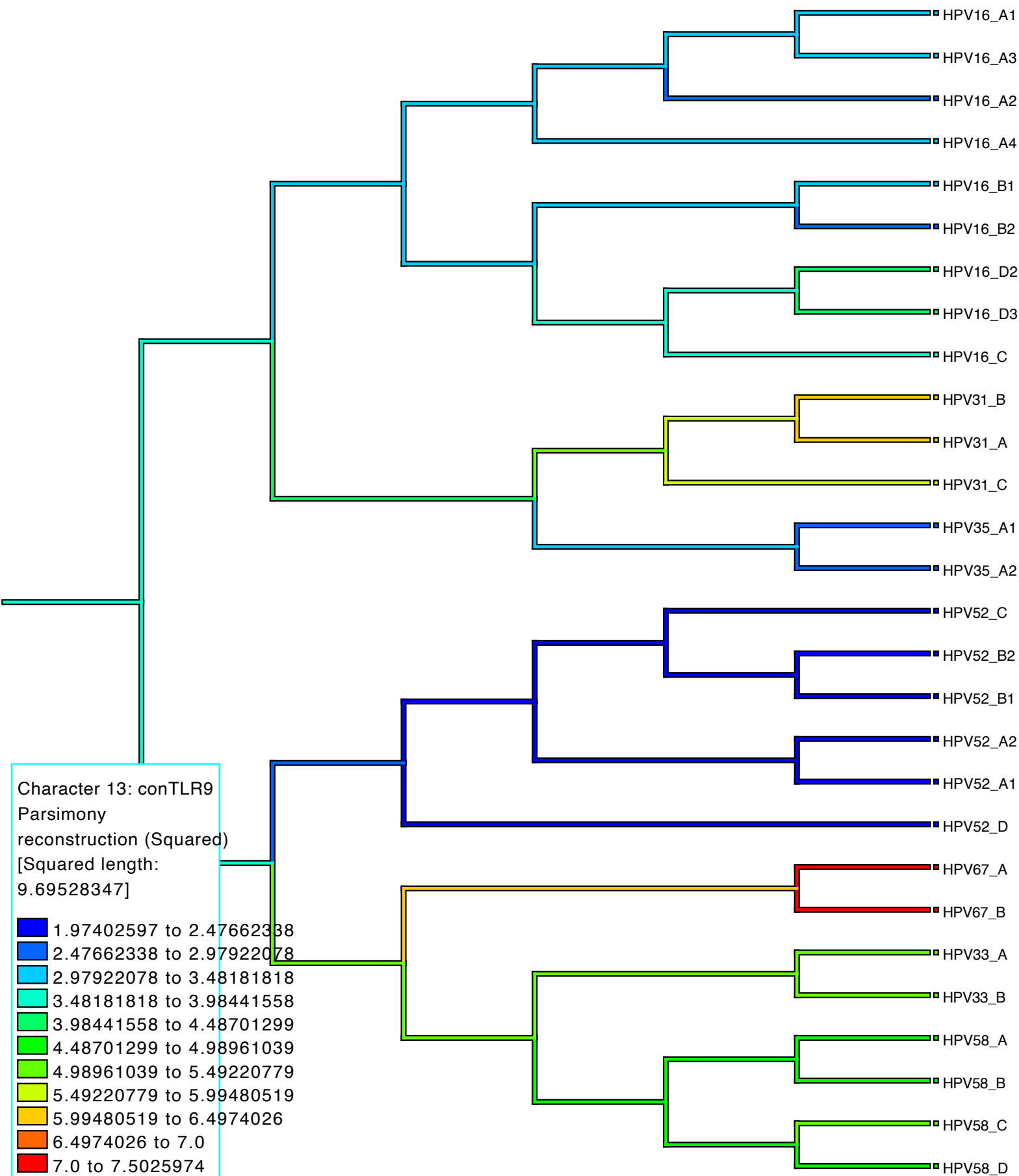

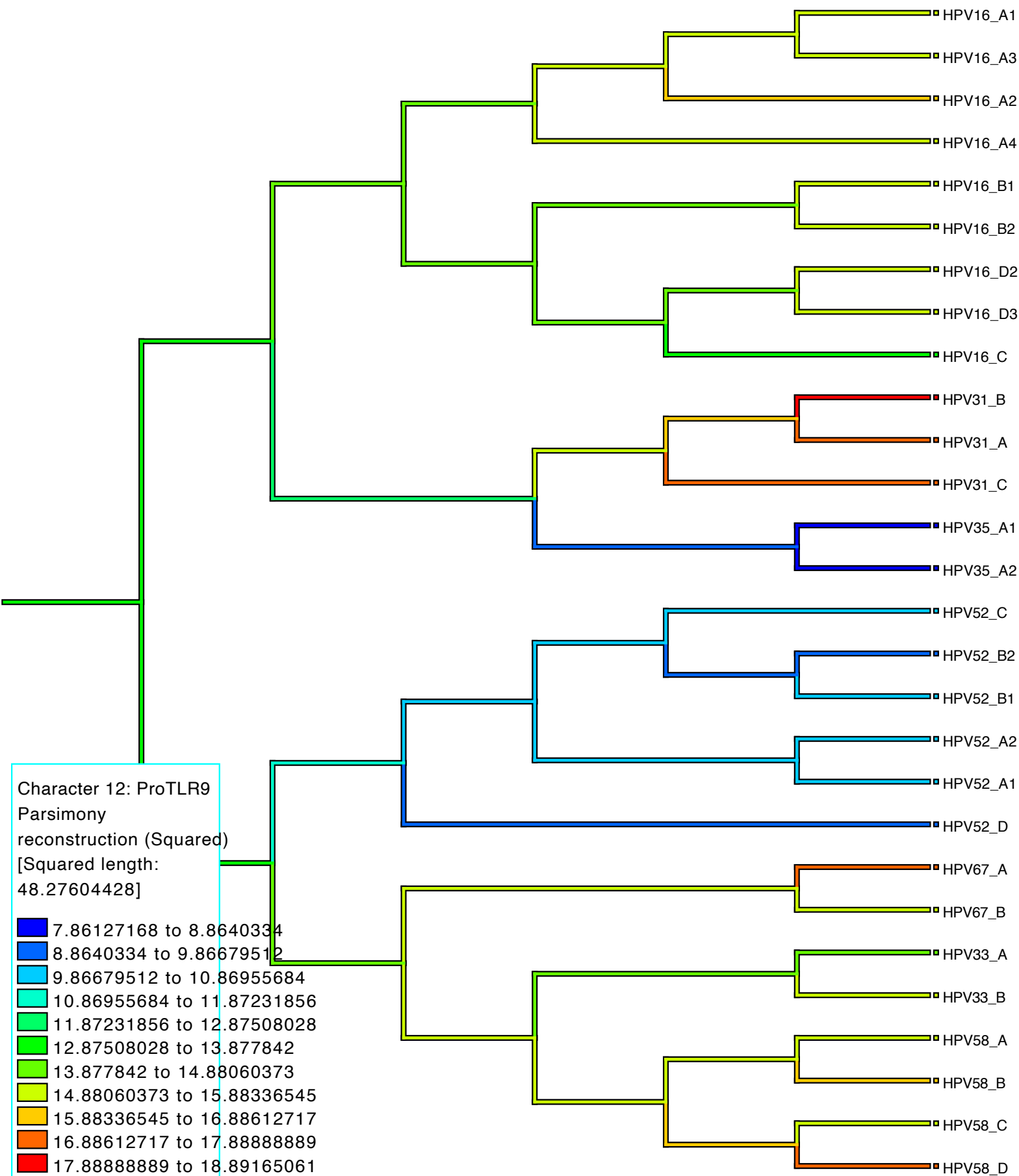

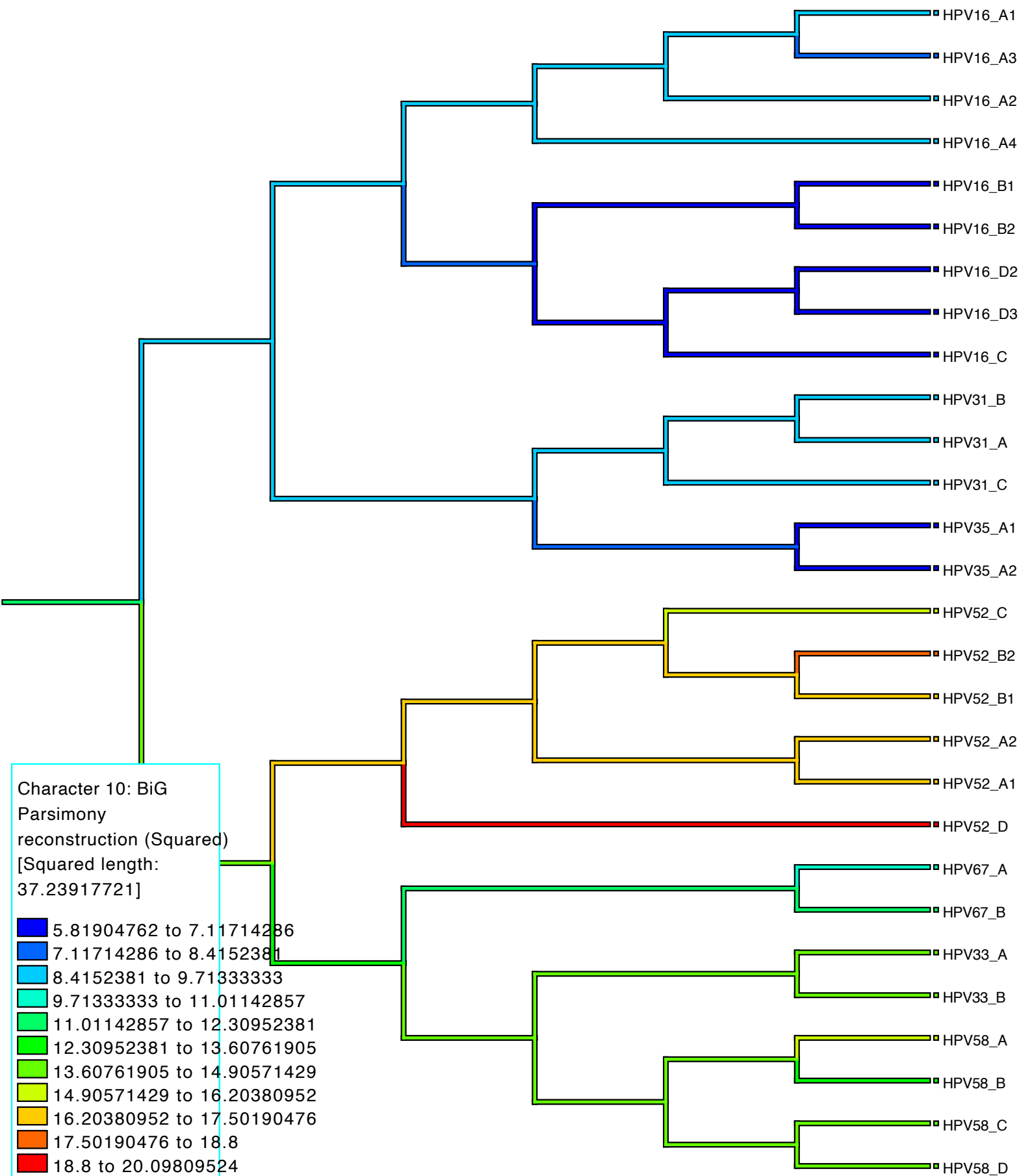

Supplement: Supplementary file 1 [file viruses-15-01631-s001.zip › S5. HPV Alpha9 Ancestral Reconstructions.pdf]
